# A Hybrid Bioprinting–Microfabrication Approach to Controllable Multitype Spheroid Arrays for Modeling Tumor Heterogeneity

**DOI:** 10.1002/adhm.71725

**Published:** 2026-09-20

**Authors:** Simone Bonetti, Eva Clemente, Emanuela Mensà, Marco de Maria, Fulvia Farabegoli, Emanuela Saracino, Barbara Bortolani, Emanuela Marcelli, Renato Brandimarti, Dario de Biase, Giampaolo Zuccheri

**Affiliations:** ^1^ Institute for the Study of Nanostructured Materials National Research Council of Italy Bologna Italy; ^2^ Department of Pharmacy and Biotechnology University of Bologna Bologna Italy; ^3^ Institute for the Organic Synthesis and Photoreactivity National Research Council of Italy Bologna Italy; ^4^ eDIMES Lab – DIMEC, Policlinico S. Orsola‐Malpighi University of Bologna Bologna Italy; ^5^ Interdepartmental Center for Industrial Research on Health Science and Technologies (CIRI‐SDV) Alma Mater Studiorum Università di Bologna Bologna Italy; ^6^ S3 Center Institute of Nanoscience of the National Research Council at the University of Modena Modena Italy

**Keywords:** 3D culture, bioprinting, cancer modeling, tumor heterogeneity, tumor microenvironment, tumor spheroids

## Abstract

The intrinsic heterogeneity of solid tumors remains a crucial barrier to understanding cancer biology and predicting therapeutic outcomes. Here, we introduce a scalable and versatile biofabrication platform that enables the controlled assembly of multitype spheroid arrays (MSA) within standard culture microplates. By performing programmable bioprinting within soft, microstructured culture devices, we achieve the precise spatial deposition of multiple cell types into defined microwells, yielding reproducible arrays of heterogeneous 3D tumor spheroids that collectively recapitulate the macro‐ and micro‐heterogeneity of solid cancers. We demonstrate that this approach permits the fine‐tuning of spheroid size, composition, and arrangement. It also supports the delayed secondary programmed seeding of cells on pre‐grown spheroids, mimicking biological processes such as the invasion of immune cells. These methods enable quantitative drug‐response profiling within a single assay. They overcome some critical limitations of current 3D cancer models, introducing the concept of the “cancer‐in‐a‐well” technology. This technology provides a simple‐to‐use, cost‐effective, high‐throughput, and highly reproducible platform to recreate the complexity of tumors in vitro using methods and instruments that can be potentially used by any researcher in any cell culture lab. We can foresee a positive impact on cancer biology studies, drug screening, and personalized oncology.

## Graphical abstract

This work introduces a “cancer‐in‐a‐well” technology that recreates tumor heterogeneity in vitro using multitype spheroid arrays. Combining bioprinting/microdispensing with microstructured culture devices, the system improves drug screening and modeling of tumor complexity. This advances 3D culture approaches beyond 2D limitations, supporting more predictive preclinical testing and reducing animal use.

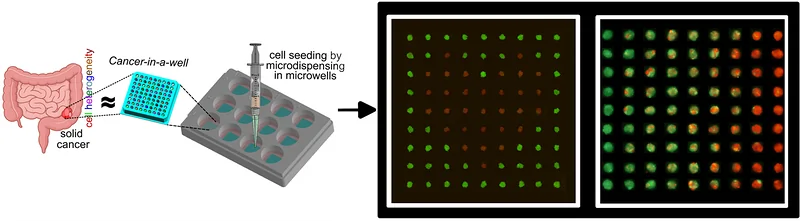

## 1 Introduction

Cancer is responsible for 1 out of 6 deaths worldwide. With the projected increase in cancer cases in the future (35 million cases expected by 2050 according to the American Cancer Society [1]), the fight against cancer remains a global priority. A multipronged approach is essential, with particular emphasis on the development of new therapies and the more effective use of existing ones.

The development and testing of new drugs require better and more advanced cell models that can accurately recapitulate or even predict a patient's cancer response to treatment. In the effort to replace and reduce the use of laboratory animals, it is essential to develop new 3D models of cancer or of entire organs and whole patients that are easy to use, relatively inexpensive, widely accessible, and capable of efficiently and rapidly testing multiple drugs.

Along the patient treatment pipeline, cancer cells often become available either through liquid or needle biopsy or via surgical resection of a solid tumor. It would be ideal (and it has been suggested and pioneered [2, 3]) that such patient cells, along with cancer genetic markers, could be used to test the susceptibility of the patient to the available chemotherapy, to provide the best possible care, limiting the side effects and the development of drug resistance.

To be most useful to the stated purpose, in vitro culture systems must replicate the complexity of the tumor microenvironment (TME), which can influence treatment outcomes by supporting tumor cell metabolism [4] or partially neutralizing therapeutic agents. Personalized in‐vitro models of a patient's own cancer cells would prove especially useful to patients with cancers with uncommon variants of uncertain significance (VUS) [5]. These are genetic variants of cancer oncogenes whose impact on the function of the protein is not known. For these patients, clinicians are often limited to using cytotoxic drugs rather than targeted and often more patient‐friendly therapies. Gaining deeper insight into the unique characteristics of each patient's cancer might open the way for more effective and personalized treatment strategies.

Common 2D cultures of adherent cells are used to model solid tumors, but often fail to capture the complex behavior of real tumors [6, 7]. Here, cells grow in a single layer, fully exposed to the surrounding medium and adhering to rigid plastic surfaces that do not resemble the natural tissue environment, failing to mimic the resistance and the complex treatment response of a patient's cancer. As a result, many anticancer drug candidates that are effective in these simplified models prove ineffective in clinical trials. As an example, drugs like cisplatin, doxorubicin, 5‐fluorouracil, ralimetinib, lapatinib, and mitomycin C represent cases where 2D cell culture testing was insufficient to predict clinical outcomes reliably, and cells in 2D cell cultures have been found to be up to 100 times more sensitive to drugs than the real solid tumor (as in the case of doxorubicin) [8]. It appears that only 5% of oncology drug candidates have succeeded in clinical trials after being tested only with traditional 2D cell culture [9]. Lately, various strategies have been proposed to overcome these limitations and better recapitulate the TME [10, 11, 12].

It has been increasingly recognized that the mechanical properties of the cell‐growth environment are highly relevant in determining cell behavior [13, 14]. Cell growth matrices have been developed and made commercially available [15, 16]. Additionally, research groups have been proposing alternative biomaterials and geometries to mimic cancer mechanics [17]. 3D cell culture has undergone rapid development due to its potential in reproducing the behavior of real tissues in vitro, together with reducing the use of lab animals. Remarkably, many cancer drugs display a significantly lower efficiency in 3D models, possibly due to a combination of slower cell growth rate, the presence of diffusion barriers, and the presence of different cell types that may affect the drug uptake [18]. It is not uncommon that a treatment that wipes out a 2D cell culture only slows down the growth of a 3D culture of the same type of cells or kills the cells in the external layers while leaving the innermost ones ready to restart the growth once the drug pressure is reduced [19]. Additionally, comparative results found that the transcriptomics of 3D models is similar to that of tissues [7].

Many 3D culture techniques are currently available [20]. Some rely on complicated, proprietary machinery, such as bioreactors, to keep the growth conditions stable and provide an environment for 3D growth. Others use relatively simple plasticware and aim at making seeding and growth operations simpler, sometimes lacking the possibility of recreating the complex TME. Within this latter group, we can list the commercial or custom‐built microwell culture devices that allow the growth of many homogeneous spheroids in the same well, and that entail seeding and growth procedures that are very similar to the classic 2D culture [19]. In these devices, a controlled cell composition of the 3D spheroids can be achieved by mixing different types of cells, obtaining a homogeneous distribution throughout the plate well. This procedure will yield heterotypical cell spheroids, which could be used to locally replicate the TME, however they will not be able to represent the heterogeneity of such microenvironment, or of the whole tumor, on a larger scale. Consequently, the complexity of the drug response of a patient's tumor will not be reproduced.

On the other end of the spectrum, high‐tech systems such as microfluidics‐enabled organ‐on‐chip or body‐on‐chip [21] let the researcher produce highly complicated and controllable systems, where both 2D and 3D cell systems may exchange chemical signals under fine control and be pharmacologically tested. Given the complex technical setting, this approach may be expensive, difficult to replicate, low‐throughput, and result in limited statistical significance and reduced usefulness.

In this manuscript, we present a technology that mimics the complexity of a solid tumor or, in principle, an entire body in vitro, providing models to study the biology of tumor heterogeneity, bodily responses, and the effectiveness of pharmacological treatments. We like to refer to this technology as “cancer‐in‐a‐well,” as it could, in principle, achieve the full cell heterogeneity of a solid cancer within a multiplate well. By pairing cell bioprinting/microdispensing technology with microstructured culture devices, we can obtain by‐design multitype spheroid arrays (MSA) that can mimic the compositional and environmental heterogeneity of solid cancers or other cell tissues. In the MSA, each potentially different spheroid is in contact with the others through the medium and thus exchanges signals and metabolites, as in the paracrine signaling within a cancer or an organ.

## 2 Results

### 2.1 Arrays of Controlled Heterogeneous Multicellular Tumor Spheroids

The aim of this study was to develop a method to reproduce the micro and macro‐heterogeneity of tumors in‐vitro and thus provide a new tool to characterize the different TMEs and their potential effects on cancer development and drug efficacy (Figure 1).

**FIGURE 1:**
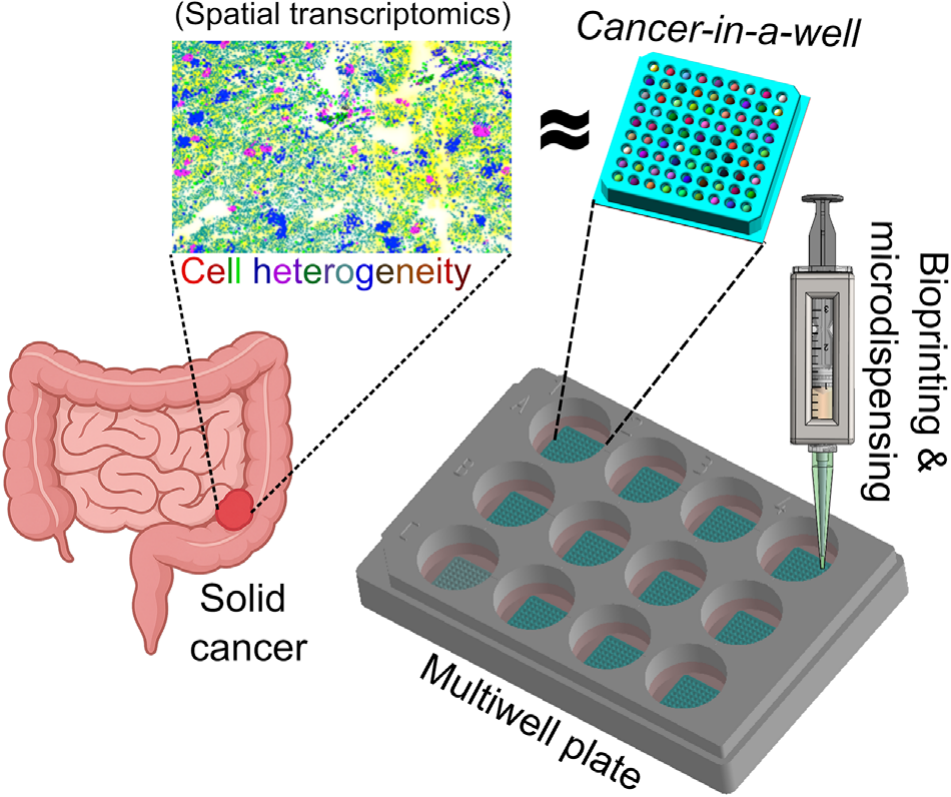
Graphical illustration of the concept of “cancer‐in‐a‐well”. The heterogeneity of a (cancer) tissue can be modeled in vitro by growing a large number of independently‐seeded spheroids/organoids that share the same medium and exchange soluble signals. Such models were prepared in a reproducible fashion thanks to the integration of bioprinting and microfabricated culture wells.

Microdevices were fabricated via soft lithography using a 3D‐printed mold. They can be designed with the appropriate patterns to emulate defined biological structures, and their microwells are sized and shaped according to the experimental goals [19]. For the aims of this study, we designed and built thin micro‐molded culture devices containing 81 cone‐shaped microwells (top diameter 1000 µm, bottom diameter 500 µm, bottom radius of curvature of 250 µm, depth 1300 µm) and placed them at the bottom of the well in a standard 12‐well microplate. To better replicate the mechanical microenvironment of a tumor tissue, the device was made of soft biocompatible materials, such as silicone rubber or hydrogel (Figures 2a; Figure S1 and S2), avoiding cell contact with rigid plastic [19].

**FIGURE 2:**
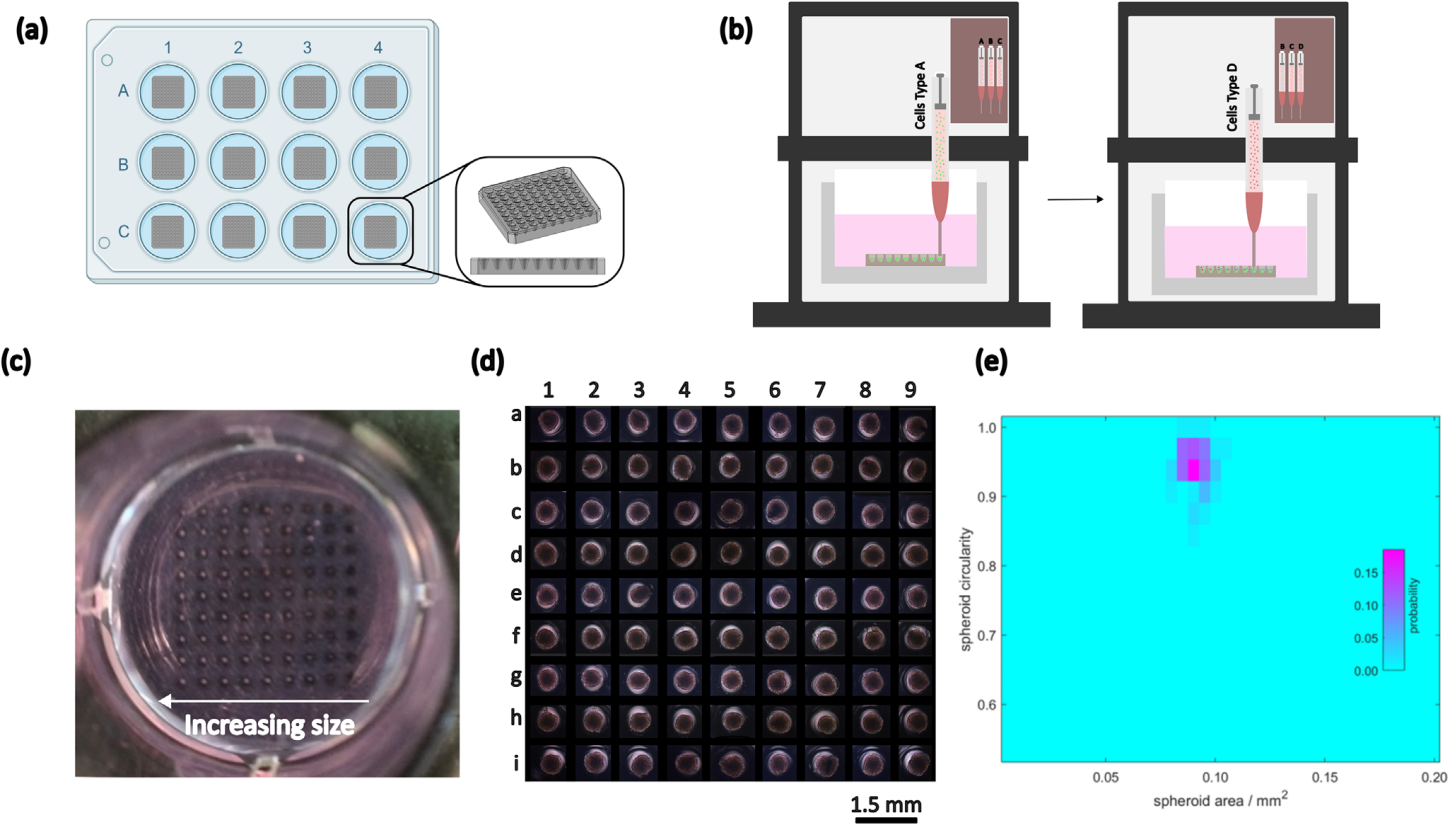
Schemes of the devices and processes of bioprinting spheroid arrays and prototypical results for a simple cell spheroid seeding. (a) Schematic representation of the plate with microstructured soft devices and [figure produced using SOLIDWORKS version 2021] (b) the printing technology [figure produced using Inkscape]. (c) Photo of a microdevice in the well containing an array of HCT116 cell spheroids with increasing size (right to left, increasing size in the columns, same in rows) after programmed seeding with linearly increasing cell numbers. (d) Composite micrograph of a device containing constant‐sized HCT116 spheroids, after seeding 320 cells per microwell and culturing for 9 days. The clear circles are the bottom parts of the transparent microwells with the darker spheroids inside (while their sides appear as dark) (e) probability‐normalized 2D histogram of the distribution of values for spheroid size (projection area) and circularity in bioprinted devices, as in Figure 2d. The *x*‐axis range was chosen to cover the possible growth size in the used device microwells.

After adding the culture medium to wet and cover all the device, each microwell was seeded with the desired number of cells using one or more tools of a microextrusion bioprinter (Figure 2b). Controlled extrusion delivers a known number of cells within the microwell; cells quickly sediment in the lower‐density culture medium and cluster on the round‐shaped microwell bottom. A controlled composition of each spheroid seeding can be achieved. At the end of seeding, the individual microwells contained the desired number and variety of cells. Thanks to culturing in a common incubator, clustered cells grow into 3D spheroids. An individual 3D multicellular tumor spheroid (MTS) grows in each microwell: this creates a multitype spheroid array (MSA) in the plate well. Within each plate well, the numerous multitype (or identical) spheroids grow in the same medium and share or exchange chemicals, also as a function of their reciprocal distance, and thus recreate a similar environment as that in a macro‐heterogeneous solid cancer.

The customized 3D bioprinter used in this study [22] is equipped with four syringes mounted on the print tools, allowing the controlled micro‐seeding of up to four different cell types, cell mixtures, or materials in each microwell of the array. The tip of the syringe needle is lowered in each microwell at a controlled height and used to deposit the cell suspension solution in a volume smaller than the microwell volume, to prevent the dispersion of cells outside the microwell and to adjacent microwells. Alternative designs of bioprinters or micro‐dispensers could be used as well.

Thanks to the developed software, after initial alignment of the tip of one syringe with the microwell array, the tip height and the dispensed volume can be set for each syringe and each microwell, and the desired number of each cell type can be dispensed in a rapid succession, to limit the time the plate spends outside the incubator. Seeding can take a time of one to two minutes per cell type per MSA (seeding a 12‐well plate with twelve full 81‐microwell devices takes about 30 min per cell type).

Figure 2 shows the MSA made of a single cell type, the human colon cancer cell line HCT116. (see Supporting Video S1 for a recording of the MSA printing process). Figure 2c shows an MSA with cell numbers growing linearly across the array from left to right. Figure 2d shows an array of HCT116 spheroids homogeneous in size and composition, after 9 days of growth in culture. This demonstrates the efficiency of the printing system and the successful formation of circular spheroids that are reproducible in size, as also shown in Figure 2e.

To produce MSAs with the desired characteristics, several parameters, and experimental conditions were initially tested. Among these preliminary studies, it was verified that the density and viscosity of the medium can affect the reproducibility and homogeneity of the microdispensing procedure. The use of culture medium alone for the HCT116 cell suspension led to a progressive, unwanted decrease in the number of seeded cells, especially in the case of consecutive printing of multiple MSA in the microplate. This was likely due to the gravitational sedimentation of the cells within the solution on the flat bottom part of the syringe during the setup and printing process.

To reduce this problem and improve the homogeneity of cell seeding and the uniformity of the resulting spheroids, the density of the medium was adjusted to 14% (*w*/*v*) of the nonionic density‐gradient medium Histodenz [23] to maintain the cells in suspension throughout the bioprinting process. Figure 3a compares the efficiency and control of dispensing cells, with and without Histodenz, from 0 to 320 cells/microwell. The number of cells was counted by direct observation under an inverted microscope. Four microwells for each condition (increasing cell number across the columns) were averaged for each MSA. The measurement was averaged for four MSAs at separate positions in a fully seeded 12‐well plate (at positions 1, 4, 8, and 12). The plot shows that the Histodenz‐containing solution allows good adherence to the calculated seeding numbers, displaying a linear increase in cell number across the columns and consistent cell counts across all four devices, as evidenced by the smaller standard deviation. The slight increase in standard deviation observed at higher cell numbers is expected and consistent with the experimental setup. Table S1 summarizes the estimated dispensing errors during cell seeding, providing a clearer overview of the bioprinter performance and the robustness of the overall procedure. The repeatable size of the spheroids seeded and grown in a full plate (12 devices) is shown in Figure S3. In contrast, the cell seeding without Histodenz shows a nonlinear relationship between seeding settings and cell counts with high variability between devices. Moreover, with non‐adjusted density medium, cells were also found in device columns where they were not intentionally seeded, suggesting that some cells were dispersed within the well. Consequently, Histodenz‐containing medium was used throughout the reported experiments to improve spatial control during deposition. We confirmed that 14% Histodenz has no detrimental effect on the viability of the HCT116 cells, if these are exposed to it during the operations (see Figure S4).

**FIGURE 3:**
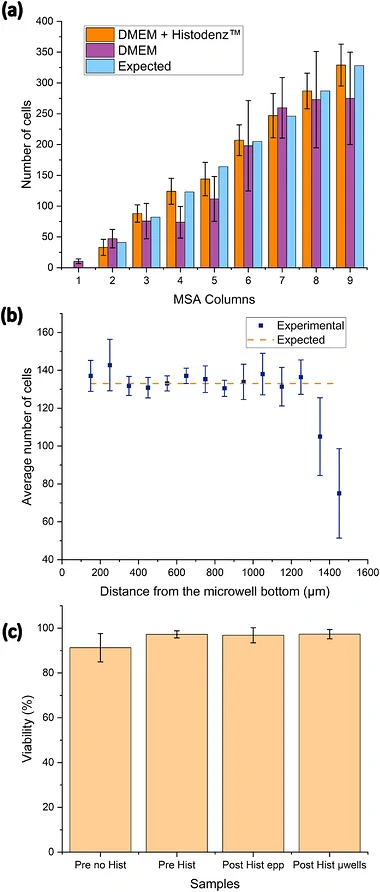
Characterization of the seeding performance with different bioprinting conditions. (a) Comparison between the expected number of cells seeded per microwell and the experimental values obtained using either DMEM medium alone or DMEM + Histodenz (*N* = 18 microwells for each condition; error bars = standard deviation (SD)). (b) Comparison of the number of cells seeded per microwell at different heights from the bottom of the microwell, ranging from 150 to 1250 µm (*N* = 18 microwells each condition; error bars = SD). (c) Live/dead staining analysis of HCT116 cells after trypsinization (Pre, no Histodenz, number of cells = 2259), after incubation in DMEM + Histodenz (Pre, with Histodenz, number of cells = 357), after extrusion in a tube (Post Hist Eppendorf tube, with Histodenz, number of cells = 370) and after extrusion in microwells (Post Hist microwell, with Histodenz, number of cells = 2512) (error bars = SD).

### 2.2 The Influence of the Extrusion Height on the Seeding Efficiency

One of the crucial operational parameters of the cell seeding procedure is the height of the tip of the syringe needle in the microwell. When printing in a pristine microwell, the height can, in principle, be regulated at will, taking into account the chosen microwell geometry. As it is possible to deposit cells at a later time on already pre‐seeded microwells (vide infra), the height of the tip should be adjusted accordingly. To optimize the printing parameters for cell deposition, different tip heights (Z position) between the needle tip and the bottom of the microwell were tested. The Z values were changed from 150 µm above the bottom of the microwell to 1450 µm, that is, at the top surface of the microwell device (which has a total height of 1300 µm). Figure 3b shows the count of the cells in the microwells after extrusion at varying Z position values, compared to the count expected considering the extrusion volume and cell suspension concentration. Within the tested Z position range (150 to 1450 µm), the count of deposited cells closely matched the expected number. At Z position values higher than 1350 µm, that is, above the surface of the device and the upper edge of the microwells, the number of cells seeded in the microwells decreased significantly as cells were also dispensed onto the surrounding device surface, with a significant worsening of cell seeding efficiency and control. When counting the printed cells, clear differences were observed between the lower Z position range (150 to 450 µm) and the higher values. At Z values below 450 µm, cells tend to distribute along the periphery of the tested microwells, forming a ring (Figure S5a) or a disordered pattern. In contrast, for Z values above 550 µm, cells were deposited in the center of the microwell, forming a compact and uniform spot (Figure S5b). This compact cell spot is preferable for spheroid formation, as optimal cell aggregation relies on direct cell–cell interactions, which are promoted by the dense, clustered cell arrangement at the bottom of the spherically‐shaped microwell. Based on these results, a Z position of 650 µm was selected for cell printing into the microwells.

### 2.3 Cell Viability After Extrusion Seeding in the Microwells

During extrusion, cells slowly translocate through a syringe needle (250 µm internal diameter). As the shear stress could impact the cell viability, the flow rate was reduced to the minimum practical value (1.4 µL/s). To evaluate the cell viability after the printing process, a live/dead assay was performed on HCT116 cells under different conditions, taking the cells immediately after resuspension from the culture flask as a reference. Viability was assessed for cells in a 14% w/v Histodenz suspension to evaluate the impact of medium density, and after extrusion through the bioprinter syringe and needle under operational conditions: immediately after extrusion or after 1 h of incubation in the microwells. As shown in Figure 3c and Figure S6, the percentage of viable cells remained high across all three conditions. The results indicate that cell viability was not significantly affected by either exposure to Histodenz or by our extrusion process (see also Figure S4).

### 2.4 Homotypical and Heterotypical MSA Seeding and Growth

To test the performance of the system and the growth of the spheroids, two different types of HCT116 MSA designs were printed: one with uniform spheroid sizes (constant size) and one with a column‐by‐column linearly increasing size gradient. Figure 4a shows representative images from rows G and H of the same device at three different time points (3, 6, and 9 days of growth) for the constant‐size MSA. Each microwell in the device was seeded with 320 cells/microwell to generate spheroids of consistent initial size. Over time, an increase in spheroid size is clearly visible, with the same dimensions observed across spheroids at the same time point, confirming their uniform growth. Figure 4b reports the monitoring of the growth and uniformity of the constant‐size MSA, confirming the high level of control of seeding and growth in the system. The values obtained at each time point across the different columns are very similar, with low standard deviations. This proves a high reproducibility of the printing process.

**FIGURE 4:**
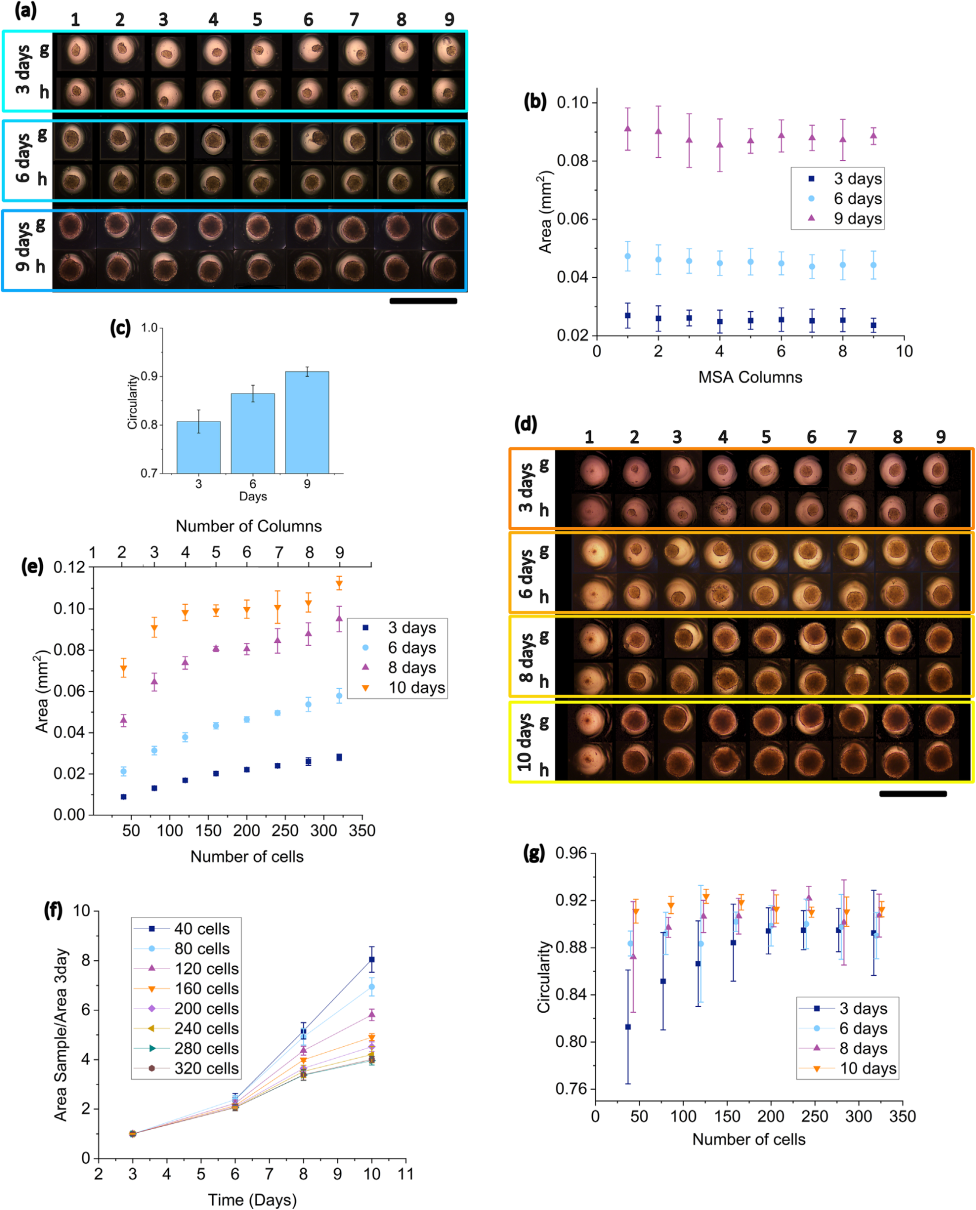
Morphological characterization of the growth of the spheroids in the microwells. (a) Two rows of the same MSA containing constant‐size HCT116 spheroids, shown at 3, 6, and 9 days (320 cells seeded per microwell) (Scale bar: 1.5 mm). (b) Average spheroid area per column for four independently‐seeded constant‐size spheroid arrays at 3, 6, and 9 days (*N* = 36 spheroid measurements per column). (c) Circularity measurements of spheroids from four constant‐size arrays at 3, 6, and 9 days (*N* = 324 measured spheroids for each condition; error bars = SD). (d) Two rows of the a MSA containing HCT116 spheroids seeded with linearly increasing size, shown at 3, 6, 8, and 10 days of growth (Scale bar: 1.5 mm). In Figure S7, the building of the composite images in panel (d) is shown as an example. For four independently‐seeded linear gradient size spheroid arrays at 3, 6, 8 and 10 days, (e) shows the spheroid average area per column (*n* = 32 each condition; error bars = SD), (f) shows the spheroid average area normalized to the 3‐day values for each condition, and (g) the spheroid average circularity.

Figure 4c shows the circularity of the spheroids, $circularity=4\pi \;\cdot \;area/circumference^{2}$ where a perfect circle has a circularity of 1. The spheroids obtained after 3 days exhibited lower and more variable circularity compared to those after 6 and 9 days of growth. Over time, the circularity of the spheroids improves and becomes more consistent across all devices.

Figure 4d shows images of the growth of two rows from a MSA device with seeded spheroids with a linear gradient of sizes (in Figure S7, the making of this composite image is shown as an example). The first column was left empty, while columns 2 to 9 were seeded with an increasing number of cells, starting from 40 cells and increasing by 40 cells per column, up to a maximum of 320 cells/microwell. The unseeded microwells remained empty and did not show any growth, while in the other microwells the spheroids grew progressively over time. The size of the microwells inherently poses limits for the growth of the spheroids, especially for those made of silicon rubber (PDMS). Figure 4e presents the monitoring of spheroid growth in MSA seeded with a linearly increasing number of cells in the wells. The spheroid area increases proportionally across the columns, reflecting the different initial numbers of seeded cells. The graph in Figure 4f illustrates how the initial number of seeded cells and spheroid size influence their growth kinetics. By calculating the ratio between the spheroid area at each time point and its corresponding area at day 3, it becomes evident that smaller spheroids grow proportionally faster than larger ones. For spheroids formed with a higher initial cell number, the growth rate tends to stabilize across different time points, likely due to limitations in nutrients diffusion within larger spheroids. Furthermore, the circularity of the spheroids is influenced by both their size and their growth over time (Figure 4g). In particular, at day 3, the circularity increases with the number of printed cells, and for all size groups, the circularity improves over time [24, 25].

To demonstrate the feasibility of using this approach to print MSA with different cell types, two variants of HCT cells were used: one stably transfected with green fluorescent protein (HCT‐GFP) and the other with mCherry (HCT‐mCherry). Each cell type was loaded into the syringe of one bioprinter tool, so the two series of microdispensing operations could be performed with minimal delay. Different MSA designs were printed. Figure 5a shows a heart‐shaped design, composed of HCT‐GFP spheroids forming the outline and HCT‐mCherry spheroids filling the inner area of the heart. Each microwell was seeded with 300 cells, and the image shows the MSA after 5 days of growth. As a second example, Figure 5b presents a chessboard pattern, with alternating HCT‐GFP and HCT‐mCherry spheroids printed in adjacent microwells. In this design, the size of the spheroids increases progressively from left to right, ranging from 20 to 230 cells per microwell (the image was taken after 5 days of growth). In both MSA designs, the printed patterns remain clearly visible, with no detectable migration of cells between adjacent microwells, confirming the robustness and fidelity of the printing process.

**FIGURE 5:**
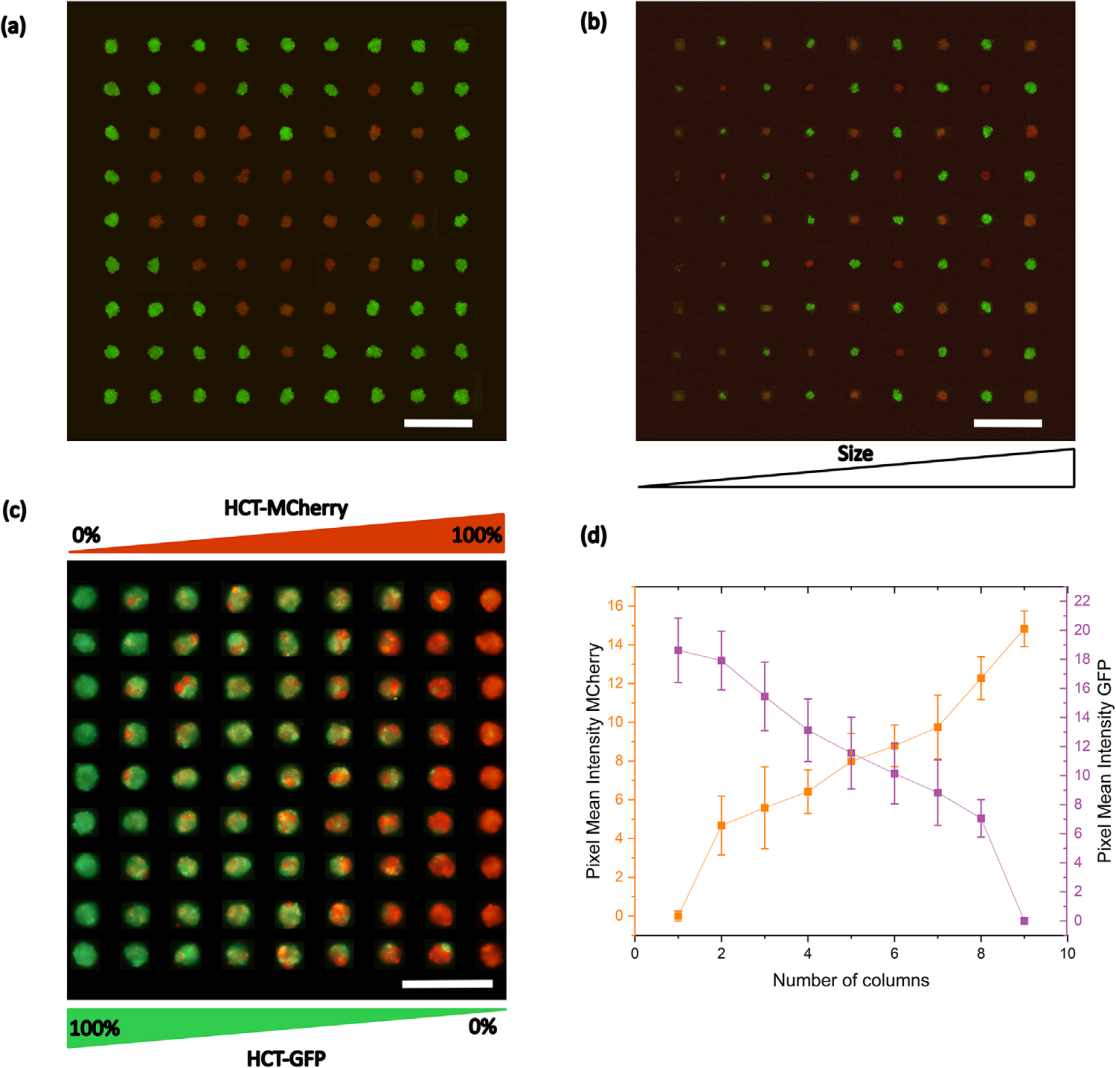
Examples of positional patterning of multitype spheroid arrays in multiplate wells. (a) A constant size MSA (300 cells/microwell, 5 days of culture) printed in a heart‐shaped pattern using HCT‐GFP cells (green) for the background and HCT‐mCherry cells (red) for the heart (Scale bar: 1 mm). (b) Two overlapping linear gradient‐sized spheroid arrays (ranging from 20 to 230 cells/microwell, 5 days of culture) printed in a chessboard pattern with alternating HCT‐GFP and HCT‐mCherry spheroids. (Scale bar: 1 mm) (c) MSA with a constant size of spheroids with a gradient cell composition of HCT‐GFP and HCT‐mCherry across the columns (from 0 to 320 cells/microwell of either type to make a total of 320 cells/microwell, 5 days of culture, scale bar: 1 mm). (d) Graph showing the mean pixel intensity of GFP and mCherry fluorescence across the columns of the array shown in (c) (*N* = 36 each condition (4 MSA devices); error bars = SD, arbitrary fluorescence units as of the camera reading).

Figure 5c shows a MSA made with a left‐right gradient composition of HCT‐GFP and HCT‐mCherry cells in constant‐sized spheroids: the cumulative number of cells seeded in each microwell was constant in the MSA (micrographs taken after 7 days in culture). For both cell types, the number of seeded cells ranged from 0 to 320 per microwell. The leftmost column contained only HCT‐mCherry cells, while the rightmost column contained only HCT‐GFP cells. The resulting MSA shows a controlled spatial distribution of the two cell populations, with size‐homogeneous spheroids across the array. This controlled pattern is further described by the mean fluorescence intensity of GFP and mCherry signals for each column (Figure 5d). The fluorescence signals of both populations follow the expected gradients, which directly correspond to the linear increase in the number of cells seeded per microwell. The graph in Figure S8 highlights the uniformity of spheroid size across four different MSA arrays, located in positions 1, 4, 8, and 12 of the 12‐well plate, where all 12 devices were prepared in the same way, further demonstrating the consistency of the printing technique when applied to multiple cell populations. In addition, by comparing the resulting spheroids areas under similar conditions shown in Figure 4 and Figure S8, it is evident that spheroids printed using the same total number of cells exhibit comparable area values across all printing strategies: the constant size, the linear gradient size, and the bidirectional gradient. These results further underscore the degree of control and repeatability of the applied biofabrication strategy.

### 2.5 Characterization of Drug Response Using MSA With Spheroids of a Range of Sizes

As a proof of concept for the application of this system, the effect of cytotoxic drugs on MSA of HCT116 was investigated. To this end, arrays of spheroids seeded with linearly increasing cell numbers (from 20 to 320 cells per microwell) were treated with 25 µM 5‐fluorouracil (5‐FU), the expected IC50 for this cell type [26] (see also Figure S9 for our experimental determination with the same cells). The bioprinted spheroids were treated with the drug for 3 days starting on day 3 after seeding [27]. The effects on spheroid size and morphology were evaluated. Figure 6a shows the analysis of spheroid areas comparing controls at 3 and 6 days and 5‐FU‐treated samples at 3+3 days. 5‐FU did not suppress the growth completely, and thus, for both cases, the areas of spheroids analyzed at day 6 are larger than those measured at day 3, while the 5‐FU‐treated MSAs showed lower growth compared to the control MSAs. The ratio between the areas of spheroids after 6 days and after 3 days was calculated as an attempt to evidence the influence of the spheroid sizes on the drug sensitivity (see Figure 6b). In the untreated samples, the growth rate is higher for smaller spheroids, as expected, evidenced in previous experiments (Figure 4f) and here confirmed. In 5‐FU‐treated spheroids, while the growth rate is expectedly lower, the size ratio day 6 /day 3 does not change appreciably over the different spheroid sizes. The difference in the ratio between treated and control conditions is, anyway, more pronounced in smaller spheroids than in larger ones. This pattern indicates that the inhibitory effect of 5‐FU affects smaller spheroids more than larger ones, possibly due to the higher cell density of bigger spheroids compared to smaller ones. Moreover, as previously shown, smaller spheroids have a higher growth rate compared to bigger spheroids, making 5FU more toxic in small spheroids, where it can penetrate the entire structure of the spheroids.

**FIGURE 6:**
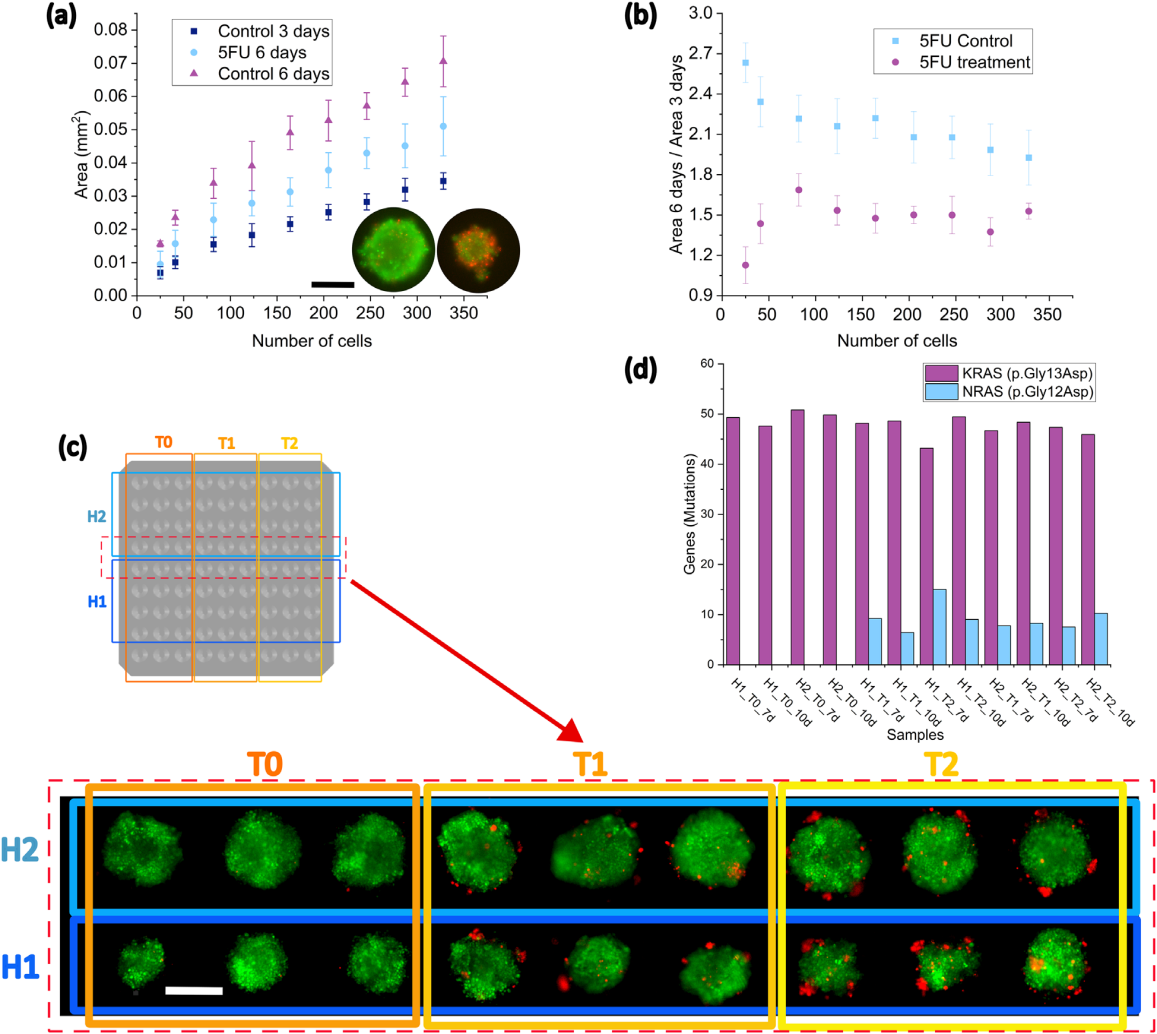
Examples of treatments of MSA with drugs or immune cells. (a) Average area of HCT116 MSA spheroids after 3 days of culture, followed by 3 days of treatment with 5‐FU. The blue markers represent the spheroid areas at day 3, before treatment. The purple marker corresponds to the untreated control at day 6, and the light blue marker shows the spheroids at day 6 treated with 5‐FU for 3 days (*N* = 36 each condition; error bars = SD). The live (green cells) and dead (red cells) fluorescence images at the bottom of the graph show examples of the 6‐day untreated (left) and treated (right) spheroids seeded with 320 cells (Scale bar: 200 µm). (b) The ratio between the area of spheroids after 6 and 3 days without treatment (light markers), and after treatment (dark markers) *N* = 36, error bars = SD. (c) A schematic representation of the microdevice illustrates the areas of primary and secondary cell seedings. The primary seedings were made with two different cell numbers (H1 lower, H2 higher) leading to two HCT116‐GFP spheroid sizes. The secondary seeding was made after 4 days with different THP‐1 cell numbers (T0 = no THP‐1, T1 lower, T2 higher) seeded onto the pre‐formed MSA spheroids. Below, fluorescence micrographs of example HCT116 ‐GFP spheroids (green) and THP‐1 (red, by pretreatment with deep‐red cell tracker dye) (Scale bar: 250 µm). Data and imaging refer to 7 days of culture (4 of spheroid culture + 3 of coculture). (d) Variant allele frequency (VAF) of two chosen gene variants specific for HCT116 (KRAS) and THP‐1 (NRAS) cells. Samples are marked according to the size of the analyzed HCT116 spheroids (H1 and H2, as in the previous panel), the amount of added THP‐1 (T0, T1 T2) and the overall duration of the culture after 4 days of HCT116 culture (7 or 10 days).

The same size‐gradient seeding and treatment protocol was executed with HCT116 spheroids and paclitaxel, an alternative chemotherapeutic drug to which the HCT116 are sensitive. The results of the treatment for the spheroids of increasing size are summarized in Figure S10. Analogously to 5‐FU, smaller spheroids appear more sensitive to the growth‐inhibiting treatment.

### 2.6 Secondary Seeding of Multitype Spheroid Arrays

The method proposed in this work also allows for subsequent individually‐tailored cell seeding operations on the device microwells, or any other treatments on the MSA with the bioprinter tools after the initial seeding and culturing. This, for example, enables the researchers to emulate biological responses of the human body to the tumor, such as the invasion of immune system cells. Alternatively, one can study the interaction of the formed MSA with different suspended cell types or materials, while maintaining the ability to create a heterogeneous architecture that better mimics in vivo conditions.

Figure 6c shows an example of this type of post‐printing, post‐growth treatment. An MSA was made of two HCT spheroid populations: H1 grown from seeding 80 cells/microwell (in rows e‐h of the MSA) and H2 from seeding 320 cells/microwell (corresponding to MSA rows a‐d). fluorescently‐labeled HCT‐GFP cells were used. After four days of growth, the MSA was treated with differentiated THP‐1 cells (macrophages derived from an immortalized monocyte line, fluorescently labeled with deep red cell‐tracking dye). To perform this procedure, the preprinted MSA plate was reinserted and realigned into the printer, and the desired numbers of THP‐1 cells were dispensed into the corresponding microwells. In the example shown in Figure 6c, the first three columns were left untouched (T0), while 40 THP‐1 cells/microwell were added to the three central columns of the MSA(T1), and 120 THP‐1 cells/microwell were added to the last three columns (T2). The micrographs in Figure 6c show typical fluorescence images of the spheroids after 7 days of incubation (4 days for HCT116 spheroids and 3 days for THP‐1), displaying one row with larger (H2) and one with smaller spheroids (H1). Green fluorescence evidences HCT116 cells, while red fluorescence shows THP‐1 cells. Analysis of the micrographs reveals that the first three columns (T0) show no red fluorescence, indicating the absence of THP‐1 cells. In contrast, the last three columns (T2) display a higher number of red fluorescent spots surrounding the green spheroids compared to the three central columns (T1), which show fewer. This confirms the capability of this method to selectively re‐seed preprinted MSA with spatial precision.

### 2.7 Spheroid Growth in a Model Extracellular Matrix

Matrigel and other extracellular matrix (ECM) hydrogels contain basement‐membrane proteins and bound growth factors that strongly influence cell behavior. They generally provide a more physiological condition to model cancer biology than growing cells free in the medium. Embedding spheroids in Matrigel can prove beneficial in better reproducing the mechanical environment of cancer and in setting conditions for testing the efficacy of drugs that are closer to the in vivo state.

In the presented spheroid seeding method, the extrusion in low‐viscosity medium is necessary to leverage gravity in forming spheroids at the bottom of microwells. It is nevertheless possible to embed the cells deposited at the bottom of the microwells or the newly formed spheroids in ECM after their seeding, without altering the design of the MSA. Matrigel can be manually layered on top of the MSA layer (see Figure S11) in order to characterize differential growth or drug response due to the presence of the ECM model. As an alternative and similar to what was reported in the previous section, a small and controlled amount of Matrigel could be extruded in the depth of the microwell, as to embed the individual spheroids. This second approach has the advantage that different amounts or different concentrations of ECM can be used for each spheroid microwell. As hardware modifications are necessary to perform this secondary extrusion, we have not tested this during this work.

### 2.8 Genetic Analysis of the MSA

Genomic techniques are crucial in characterizing the validity of the MSA models and in comparing them with real cancers. While single‐cell genomics can be performed routinely by many research labs nowadays, our ability to carefully pick individual spheroids from the MSA without disrupting their cell architectures still needs to be fully developed. Each grown spheroid is made of hundreds to thousands of cells: it is important to verify that this biomass is enough to be characterized with genomics techniques such as those in use in the clinical laboratories that routinely characterize tumor biopsies. To verify this, a small number of spheroids were withdrawn from the secondary‐seeded MSA described above and subjected to NGS analysis of gene mutation variants. Interestingly, it was found that one to a few spheroids provided enough cells to complete the analysis in full compliance with the quality control.

By NGS, 5 different mutations were detected when both HCT116 and THP‐1 cells were present in the spheroids: *CTNNB1* p.Ser45del, *PIK3CA* p.His1047Arg, *MET* p.Leu238TyrfsTer25, *KRAS* p.Gly13Asp—all characteristic of HCT116—and *NRAS* p.Gly12Asp, expected in THP‐1 cells. No *NRAS* p.Gly12Asp variant was found in the HCT116‐only spheroids. The frequencies of specific allelic mutations (VAF) give information on the spheroid cellular composition and could be used as an alternative to other techniques to track changes during MSA culture, especially in non‐fluorescently labeled arrays. The frequencies of detected allelic mutations of our spheroids are reported in Table S2 and two key ones are plotted in Figure 6d.

## 3 Discussion

All tissues in the human body are heterogeneous, exhibiting spatial variations, deeply impacting their functionalities [28]. Even simple organs are made of scores of different types of cells with zonal organization. Solid tumors exhibit a vast degree of spatial variation. In the clinic, patient variability and pharmacological treatments add to this spatial and compositional complexity. The rising awareness and the AI‐enhanced abundance of pathology data are reinforcing the perspective that solid cancers are not wholly disorganized tissues, but they self‐organize in functional fractions, forming sub‐TME or other zonal organizations [29]. In this developing context, cellular models of cancer in use in laboratories are generally lacking the flexibility to recapitulate the complexity of the different TMEs and sub‐TMEs, the relationships amongst distant tissues and organs, and the macro‐heterogeneity found in late tumors.

To address this, we propose a technology to generate different TMEs that coexist within the same model. In particular, our tissue‐engineering approach enables the production of diverse and finely tunable spheroids, capable of mimicking the heterogeneous TMEs that coexist within a real solid tumor. In such a complex tumor model, the spheroids can exchange chemical signals, depending also on their reciprocal distance, which in turn can affect their growth, viability, and differentiation. Moreover, the system allows the selective processing of each TME with secondary interventions at successive time points after MSA fabrication. This can thereby emulate possible physiological responses to the tumor, such as those of the immune system, localized changes in the chemical landscape, or variations of the compositional or mechanical properties of the matrix. To enhance the statistical robustness of the assay, multiple wells in a 12‐well plate can be seeded using same protocols. Alternatively, wells can be arranged with different configurations, allowing the entire microplate to represent a miniature version of a patient's body. Throughout the culture period, it is also possible to transfer conditioned medium from one well to another, simulating long‐range interactions such as the systemic influence of cancer on distant organs or tissues. Such a procedure can be performed manually by one operator or arranged with simple timer‐controllable pumps [30].

Thanks to the user‐friendly programming of bioprinters, the seeding of such complex systems is not significantly more difficult or time‐consuming than standard cell seeding, and can be performed using commercially available high‐end bioprinters or lower‐cost models with some technological upgrades. After setting up the equipment, the customized seeding of a 12‐well plate with one microdevice per well is fully automated and can take as little as 30 min: not much longer than manual seeding and usually not long enough to affect the viability of the cells. After bioprinter seeding, the 3D cell cultures can be maintained in a standard incubator and analyzed using a wide range of real‐time or endpoint techniques [19]. The proposed methodology, while being cost‐effective, also demonstrates high reproducibility when using different types of printing protocols (for example, constant size spheroids, linear size gradient, linear composition gradient with constant size), with comparable spheroids exhibiting consistent morphological characteristics over time across the different experimental sessions and cell generations. Importantly, the developed methodology effectively prevents cross‐contamination between microwells within the same MSA, thereby preserving the intended composition of individual spheroids in each microwell. Specifically, no cells were observed in the microwells intentionally left empty in the linear gradient size MSA shown and analyzed in Figure 4, despite cells being seeded in the adjacent microwell column. Moreover, no mixing between mCherry and GFP‐labeled HCT 116 cells was detected at the pattern edges illustrated in Figure 5a,b, nor in the side columns (entirely red or green) of the MSA shown in Figure 5c. The differentiated THP‐1 cells secondarily seeded in the MSA (Figure 6c) were absent in the T0 printing condition, further confirming the lack of unintended cell migration. Overall, the results described in this report show that our technology addresses several critical aspects affecting other state‐of‐the‐art available methods.

As reported in the literature, microextrusion bioprinting is widely used to generate spheroids and tumor models [31]. However, most approaches still present important limitations. They typically produce only one spheroid per well, which prevents the study of heterogeneous structures that mimic the coexistence of different TMEs within the same culture system. Although some approaches allow the production of structures composed of multiple spheroids, they still cannot finely tune spheroid composition by mixing different solution volumes during the printing process, nor do they permit independent, secondary treatments of single spheroids. Zhao et al. [32] developed a hybrid system combining bioprinting with the hanging‐drop technique, in which a defined bioprinted volume is seeded into drops suspended above each well. This method generates samples composed of single or paired spheroids for each well. Similarly, Larger and collaborators [33] produced heterogeneous TME models using bioprinting, printing each one into wells of a 96‐well plate. Following the same principle, Utama and coworkers [34] first built individual microwells in each well of a 96‐well plate using a microextrusion technique and subsequently seeded them with cells to produce individual spheroids. Finally, Nguyen and coworkers [35] generated heterogeneous single microtissues, one per well, to mimic the TME. While valuable for specific studies, these approaches did not achieve the (spatially controlled) cohabitation of different types of TME structures within the same system.

Other studies have focused on printing different spheroids together within the same hydrogel bath, with the aim of increasing throughput, minimizing space requirements, and exploring innovative biomaterials. Bhattacharjee and coworkers [16] demonstrated the potential of Carbopol as a support matrix for bioprinting 3D structures, by extruding homogeneous cell cultures inside the material. Their work investigated cell motion and proliferation, spheroid expansion, and molecular transport within the Carbopol hydrogel. Similarly, Wei and collaborators [36] developed a novel dot bioprinting system using collagen with sacrificial gelatin dispersed in GelMA as a support matrix. These authors printed structures named SMA (spherical multicellular aggregates), which included either “mono‐spheroids,” heterogeneous spheroids designed to mimic specific TME types, or “desmoplastic spheroids,” composed of two different spheroids within the same structure, one representing the tumor and the other the stromal tissue. It appears that their approaches do not demonstrate the ability to finely tune the composition of individual spheroids within the entire printed structure. In particular, multiple overlapping injections into the support material at seeding time may deform previously deposited droplets, leading to the formation of cell aggregates with a distorted shape and a polarized structure. These phenomena hinder the formation of spheroids with a spherical shape and a polarized organization of different cell types. In contrast, our technology enables precise control over the features of each spheroid within the MSA, by dispensing defined quantities of different solutions from separate printing tools into the same microwell, as illustrated in Figure 5c. Moreover, the strategies proposed by Bhattacharjee [16] and Wei [36] do not permit subsequent processing of the spheroid array, such as the targeted introduction of immune cells or doped hydrogels into specific spheroids within the printed structures. Any attempt at selective secondary processing would compromise the structural integrity of the printed construct. These two features are crucial for engineering models that reproduce the coexistence of distinct TME types, thereby providing tumor models that more closely resemble in vivo conditions. In our proposed technology, the use of the bioprinter tool to organize a thin layer of ECM or any hydrogel on top of the spheroids still leaves the possibility open to seed additional cells later without disrupting the spheroids’ structure, allowing them to infiltrate the 3D culture.

In the last five years, aspiration‐assisted bioprinting has been developed and widely used to fabricate complex structures [37, 38]. This technology enables precise X, Y, and Z positioning of prefabricated tissue spheroids within a supporting bath. Kim's group [39] proposed a high‐throughput bioprinting approach aimed at reducing printing time and producing large‐volume 3D models, using a 4 × 4 needle array to simultaneously aspirate multiple spheroids and then position them within the support material. In this context, the MSA developed with the technology herein proposed could represent an ideal biological building block for high‐throughput bioprinting, as it allows predefining the complex X–Y structure already during MSA fabrication.

NGS analysis has demonstrated that it is possible to routinely monitor the cell populations within spheroids or their genetic drift. In fact, thanks to the ability to identify the variant allele frequencies of genes, it will be possible to test the efficacy of various drugs by monitoring changes of variants within the spheroid.

This kind of technology enables different applications. One of them is the possibility to selectively treat microwells with various types of hydrogels (e.g., Matrigel or Carbopol), enhancing the screening capabilities and the adherence to the physiological state of the proposed model. This approach allows the development of distinct TMEs within different matrices, coexisting in the same device. Alternatively, by using the same bioprinter after the fabrication of the MSA, the possibility is open to print different bioinks directly onto the device, with varying geometries and thicknesses. This strategy can enable the inclusion of biological barriers such as the blood‐brain barrier, dermal, intestinal, blood‐CSF, or placental barriers, significantly improving in vivo‐like conditions. As a result, it creates the possibility to perform drug screening on different TMEs within the tumor, each under a selective barrier. Furthermore, treatments could be applied either to all spheroids within the MSA (as exemplified in this paper) or selectively to specific spheroids by incorporating drugs into hydrogels and delivering these locally to individual spheroids or groups of spheroids in the MSA. Following the same strategy, it would also be feasible not only to test conventional drugs but also to selectively treat patient‐specific MSAs with immunotherapy‐based approaches.

In clinical oncology, approximately 20% of patients do not respond positively to standard treatments, often due to the presence of uncommon genetic pathways in their tumors [40]. For this reason, the technology presented in this study could be used to preliminarily test standards of care on MSA devices made of patient‐derived cells extracted from different areas of the resected tumor mass. This approach could provide early indications of treatment efficacy within a few weeks, rather than the 6–12 months of a patient's response. In particular, it could enable rapid testing of the effects of targeted therapies in the presence of VUS (variants of uncertain significance) or ESCAT II variants. In this way, it could be possible to provide a rapid response as to whether a patient can be treated with a particular drug that would currently be considered off‐label for that specific neoplasm, allowing the best possible targeted therapy to be tailored to the patient based on the molecular alterations identified in the tumor model.

We trust that developing advanced 3D cancer models that can recapitulate the organization of different interacting TMEs might prove useful to understand and better cure cancer, as it now appears more and more evident [41]. Once fully developed and made available, easy‐to‐use technologies like ours should make these models accessible to the global biomedical community.

## 4 Conclusions

This study introduces a flexible biofabrication strategy that couples programmable microextrusion printing with soft microstructured culture devices to generate MSA that recapitulate both the micro‐ and macro‐heterogeneity of solid tumors in vitro. The platform allows different TMEs to coexist within the same culture system, while preserving high reproducibility, throughput, and compatibility with standard incubators and imaging or analytical workflows. The ability to modulate mechanical context, embed spheroids in ECM‐like matrices, and integrate multiple cell populations—including immune cells—supports a more physiologically relevant modeling of tumor biology and drug response than conventional 2D or single‐spheroid approaches.

Beyond providing a tool for basic studies on tumor organization, immune interactions, and clonal evolution, this “cancer‐in‐a‐well” concept lays the groundwork for multiwell layouts that approximate organ‐level or body‐like architectures, including long‐range communication via conditioned media. In future developments, combining this platform with advanced readouts such as high‐content imaging, multiplexed molecular profiling, and patient‐derived samples may enable more predictive drug screening and personalized oncology workflows, ultimately helping to bridge the gap between preclinical models and clinical outcomes.

## 5 Experimental Section/Methods

### 5.1 Bioprinting Setup

The cells were micro‐seeded into each selected microwell using an extrusion 3D bioprinter based on the E3D motion system and tool changer, like those described in the literature [22, 42]. Equipped with four independent tools, the bioprinter allows for the processing of up to four different cell solutions. To complete the calibration procedure, a camera (Bottom Camera) was integrated to capture the precise position of the microwell located in the upper‐right corner of the device placed in the first well. Additionally, a support plate with an interchangeable holder, adaptable to different microwell formats, was incorporated to enhance stability during printing. Detailed sketches of all designed components are available [43]. The parts were designed using SolidWorks 2021 and fabricated using various methods, including 3D printing. The printed components were made of polylactic acid (PLA, 3D Verbatim PLA) using a Prusa MK3S (Prusa, Prague, Czech Republic). The E3D motion system configuration file developed for this application, which can be uploaded to the Duet 2 board (Duet3D), is available for download on GitHub [43]. The positioning repeatability of the tool‐changer system is shown in Figure S12.

### 5.2 Microstructured Multiwell Fabrication

Microstructured wells were created by fitting thin microstructured devices onto the bottom of commercially sourced 12‐well plates (Falcon 353043) using a custom‐designed alignment tool (Figure S1a). The microstructured culture devices were fabricated using either poly‐dimethylsiloxane (PDMS, Sylgard184, 10:1 ratio of base to curing agent, DOW Chemical Company) (Figures S1b and S2a) or agarose gel (Sigma–Aldrich) (Figures S1b and S2b). The microstructured devices were replica‐molded from microstructured resin molds. The positive mold surface was patterned as a grid arrangement of 81 micro‐cones, each 1.3 mm in height, 1.5 mm apart, with a base diameter of 0.5 mm (Figure S1b). The final device dimensions were 15 × 15 × 1.5 mm (W × L × H), with a 0.5 mm gap between the bottom of the microwells and the well floor. The molds were fabricated via 3D printing using a Stratasys J720 Dental printer with Vero Clear base material and SUP706B support material. After printing, the parts were washed using a high‐pressure water jet (Balco UK Powerblast) and treated with 7% NaOH for 30 min to remove any support material.

For PDMS device fabrication, PDMS was poured over the molds, degassed under vacuum for 10 min, and then covered with a glass slide to create a flat base. The devices were cured at 60°C for at least 120 min. Agarose microstructured devices were fabricated similarly using the same 3D‐printed molds. For this, sterile 3% agarose (Sigma–Aldrich) in 1× phosphate buffered saline (PBS, *w*/*v*) at ∼60°C was poured onto the molds, and a glass slide was placed on top to ensure a flat surface. Both types of devices were peeled off the molds, and any excess silicone or agarose was trimmed with a scalpel. To correctly position the devices within the multiwell plate, a custom‐designed manual alignment tool was used (Figure S1). While PDMS devices adhered strongly to the bottom of the wells, agarose devices required additional fixing by filling the sides with warm liquid agarose. To avoid the formation of air bubbles in the PDMS devices during the culture, the plate was stored in a vacuum chamber overnight. To sterilize the devices, the wells were rinsed with 1 mL of 70% ethanol and left for 60 min at room temperature, followed by two washes with 1 mL of sterile PBS. The wells were then left overnight in PBS to ensure complete removal of any residual ethanol from the device material. Before seeding cells, the devices were washed once with PBS, once with DMEM (Dulbecco's Modified Eagle Medium High Glucose) (Euroclone, Milan, Italy) and finally, incubated in complete medium before printing.

### 5.3 Microseeding Software

A dedicated software for microseeding the device was developed in LabVIEW. This software generates G‐code to control the bioprinter during the microseeding procedure, based on parameters selected by the operator. The tool, fully described in the Supplementary Materials, features a GUI (Figure S13) where the operator can choose the wells to process, the type of seeding, and the micro seeding and calibration parameters. The software is available for download on GitHub [43].

### 5.4 Bioprinter Cleaning and Maintenance

Before printing the multitype spheroids array (MSA), the walls, tools, and plate of the bioprinter were cleaned with 70% ethanol (with special care for the area of the plate that comes into contact with the tip). The residual ethanol was allowed to evaporate from the plate surface.

### 5.5 MSA Printing

The theoretical number of cells seeded in each well (*N*) can be calculated from the inner radius of the syringe barrel (*r* in mm), the cell concentration in the suspension (*C*, in cells/µL), and the travel distance of the plunger during extrusion (*d*) used during seeding, using the following formula:

$$N=\left(\pi r^{2}*d\right)*C$$

Different printing approaches were tested to evaluate the viability of the printed cells and the development of the printed MSA over time.

The protocol used to print monotypical and multitypical MSA spheroids is described in the Supporting Information under the Sections 1.1 and 1.2 “MSA printing with multiple syringes” (monotypical MSA) and “MSA printing with one syringe” (multitypical MSA).

### 5.6 Printing Solution Preparation

To maintain the accuracy of the micro‐seeding device in all the microwells and prevent cell sedimentation in the syringe during the printing process, 14% w/v of the nonionic density gradient medium Histodenz (Sigma–Aldrich) was added to the culture medium. The solution containing Histodenz and culture medium was vortexed for 1 min to allow complete Histodenz solubilization. Afterward, the solution was filtered with a 0.22 µm sterile filter to sterilize the printing solution. Finally, the solution was added to the pellet containing the correct number of cells and transferred into the syringe.

### 5.7 Cell Lines, Plasmids, and Transfection Experiments

For these experiments, HCT‐116 (ATCC # CCL‐247) and THP‐1 (ATCC TIB‐202) cell lines were used. The cells were cultured in DMEM High Glucose (Medium T1) and Roswell Park Memorial Institute (RPMI, ECB9006L, Euroclone, Milan, Italy) (Medium F12), respectively, with the addition of 10% fetal bovine serum (FBS) (ECS5000L, Euroclone, Milan, Italy), 1% penicillin‐streptomycin (P/S) (ECB3001D, Euroclone, Milan, Italy), and 1% L‐glutamine (L‐glu) (ECB3000D, Euroclone, Milan, Italy). In order to reliably identify and monitor the distribution of different cells in the heterogeneous spheroids, we genetically labeled the cells for seeding with reporter genes driving the expression of green fluorescent protein (gfp) and mCherry. Integration into the genomes was driven by the transposase enzyme expressed in the SB100x plasmid (SB100X in pCAG globin pA was a gift from Mark Groudine, Addgene plasmid # 127909 [44]; RRID:Addgene_127909) [45], with donor sequences inserted in the modified plasmids SBUbg and SBUbr, for the expression of gfp and mCherry, respectively. Both plasmids were derived from pSBtet‐Pur (a gift from Eric Kowarz, Addgene plasmid # 60507 [46]; RRID:Addgene_60507) [47]. The tetracycline responsive element in pSBtet‐pur was replaced by the human ubiquitin promoter from pUltraHot (a gift from Malcolm Moore, Addgene plasmid # 24130 [48]; RRID:Addgene_24130), to constitutively drive the expression of the downstream reporters. All genetic arrangements were assembled using the Gibson assembly strategy, with enzymes purchased from New England Biolabs.

One day before transfection, HCT‐116 cells were seeded in a six‐well plate at a density of 5.5 × 10^5^ cells per well, following the Lipofectamine 3000 standard protocol (ThermoFisher Scientific, MA, USA). Transfection was performed using 3 µg of either SBUbg or SBUbr plasmid along with 0.3 µg of SB100X, in DMEM High Glucose (DMEM‐HG) with L‐glu but without FBS and P/S. The selection of transfected cells was carried out by adding 0.5 µg/mL puromycin to DMEM‐HG, and after one week in culture, cells were maintained with a reduced puromycin concentration of 0.05 µg/mL. For coculture experiments involving HCT‐116 GFP and THP‐1, DMEM‐F12 (Euroclone, Milan, Italy) was used, supplemented with 10% FBS, 1% P/S, and 1% L‐glu. Prior to coculture, THP‐1 cells were stained using CellTracker Deep Red (ThermoFisher Scientific, MA, USA) following the manufacturer's protocol. The stained THP‐1 cells were then seeded onto a poly‐dimethylsiloxane (PDMS) thin layer in a Petri dish and differentiated into macrophages by adding phorbol 12‐myristate 13‐acetate (PMA, Focus Biomolecules, USA) to RPMI for 24 h (the PDMS substrate facilitates the post‐differentiation cell detachment). After differentiation, RPMI with PMA was replaced with RPMI or DMEM‐F12. For an additional HCT‐116 transfection experiment, pSBtet‐Pur (Addgene 60507) was further modified by inserting the H2B coding sequence from plasmid 11680 (Addgene).

### 5.8 Live/Dead Assay

Staining was performed with fluorescein diacetate (FDA, Sigma–Aldrich, 8 µg/mL) and propidium iodide (PI; Sigma–Aldrich, 2 µg/mL) diluted in cell culture medium without FBS. FDA labels the viable cell cytoplasm in green, and PI labels the nuclei of dead cells in red. Four types of samples were prepared and analyzed: (a) after trypsinization, cells were resuspended in 300 µL of medium containing FDA and PI; (b) after trypsinization, cells were resuspended in a solution of Histodenz at 14% *w*/*v* in cell medium, incubated at 37°C for 30 min and treated with dyes; (c) cells were resuspended in a solution of medium and 14% *w*/*v* Histodenz, printed directly into a 1.5 mL tube and treated with dyes. All three samples were incubated in the dark at 37°C for 5 min after dyes were added to the solution before fluorescence microscope analysis (Nikon Eclipse Ti microscope). (d) cells were resuspended in a solution of Histodenz at 14% *w*/*v* in cell medium, printed in the microstructured devices, and treated with dyes. The plate was incubated in the dark at 37°C for 1 h before fluorescence microscope analysis. Pictures were acquired with a 4X objective. All images were acquired by using separate fluorescence filter sets (FITC and TRITC) and merging the images. Live cells were labeled directly while in the microstructured device, after removing most of the culture medium. Staining was performed with fluorescein diacetate (FDA, Sigma–Aldrich, 8 µg/mL, ex/em 494/521 nm) and propidium iodide (PI; Sigma–Aldrich, 2 µg/mL, ex/em 536/617 nm) diluted in cell culture medium without FBS. FDA labels the viable cell cytoplasm in green, and PI labels the nuclei of dead cells in red. The double staining was performed by incubation in the dark at 37°C for 1 h before epifluorescence microscope analysis (Nikon Eclipse 80i with Hamamatsu Flash 4 sCMOS digital camera). Four different device areas were acquired with a 5X objective (Nikon, 0.15 NA) with at least 20 spheroids each, in order to analyze more than 60 spheroids for each condition. Images were captured by acquiring Z‐stacks of the samples using FITC (FDA) and Cy3 filter sets (propidium iodide) and then merged. Data visualization and statistics were obtained with Origin Pro v. 2023 (OriginLab Corporation, MA, U.S.A.).

### 5.9 MTT Assay

Cells (2000 cells/well) and spheroids were plated in triplicate in a 96‐well plate and after 48 h, treated with 5‐FU at defined concentrations for 72 h. Cell viability was measured using an MTT (3‐(4,5‐dimethylthiazol‐2‐yl)‐2,5‐diphenyltetrazolium bromide) assay [49]. The results were expressed as a percentage of treated on untreated cells (CTR).

### 5.10 Brightfield and Fluorescence Microscopy

The images were captured using a Nikon Eclipse Ti microscope, equipped with appropriate fluorescence filters, 4, 10, and 20X magnification, and a Digital Sight DS‐U3 camera.

### 5.11 NGS Analysis

DNA was extracted using the MasterPure Complete DNA and RNA Purification Kit (Lucigen). DNA was then used for the library preparation. Briefly, about 10 ng of DNA was amplified using a laboratory‐developed NGS panel designed in the Molecular Pathology Laboratory of Solid Tumors—IRCCS Policlinico di S.Orsola, allowing the analysis of the following genes (330 amplicons, 21.77 kb, human reference sequence hg19/GRCh37) [50]: BRAF (exons 11, 15), CTNNB1 (exons 3, 7, and 8), DICER1 (exons 10, 21, 26, 27, and 29), DPYD (exons 11, 13, 22, chr1 intronic regions: g.98187018‐ 98187098, g.98045419‐ 98045499 and g.97915570‐97915789), EGFR (exons 18, 19, 20, and 21), EIF1AX (exons 1, 2, and chrX intronic region g.20148634‐20148745), GNA11 (exons 4 and 5), GNAQ (exons 4 and 5), GNAS (exons 8 and 9), H3F3A (exon 1), HRAS (exons 2–4), IDH1 (exon 4), IDH2 (exon 4), KIT (exons 8, 9, 11, 13, 14, and 17). KRAS (exons 2–4). MED12 (exons 1 and 2). MET (exons 2, 14), NRAS (exons 2–4). PDGFRα (exons 12, 14, and 18), PIK3CA (exons 8, 10, and 21), PTEN (exon 5), RET (exons 5, 8, 10, 11, 13, 15 and 16), RNF43 (exons 2–10), SMAD4 (exons 2–12), TERT (promoter region, Chr5 g,1295141‐1295471), TP53 (exons 2–11), TSHR (exons 1–10), and VHL (exons 1–3). Amplified libraries were diluted to 22 pM and pooled together on the automatic platform Ion Chef by adopting Ion 510 & Ion 520 & Ion 530 Kit – Chef (Thermo Fisher Scientific. Waltham. USA) following manufacturer protocol. Finally, libraries were automatically loaded into Ion 530 Chip to be analyzed on the Ion Gene Studio S5 Prime System platform (Thermo Fisher Scientific). Data analysis was performed by adopting Torrent Suite 5.20 (Thermo Fisher Scientific). Only nucleotide variations detected in both strands were considered for the mutational calls [51].

## Author Contributions

**Simone Bonetti**: conceptualization, investigation, funding acquisition, writing – original draft, methodology, software, writing – review & editing, visualization, project administration. **Eva Clemente**: writing – review & editing, investigation. **Emanuela Mensà**: investigation, writing – review & editing. **Marco de Maria**: investigation, writing – review & editing. **Fulvia Farabegoli**: funding acquisition, validation, writing – review & editing. **Emanuela Saracino**: funding acquisition, writing – review & editing, methodology. **Barbara Bortolani**: investigation, methodology. **Emanuela Marcelli**: investigation, methodology. **Renato Brandimarti**: methodology, investigation, writing – review & editing, funding acquisition. **Dario de Biase**: funding acquisition, investigation, methodology, writing – review & editing. **Giampaolo Zuccheri**: conceptualization, investigation, funding acquisition, writing – original draft, writing – review & editing, visualization, methodology, project administration, supervision.

## Funding

PRIN PNRR 2022–20224KF8HE BASICPROMED and Progetto Metodi Alternativi 2022 of the University of Bologna, as of decreto 25 Giugno 2021 of the Ministry of Health of Italy.

## Conflicts of Interest

The authors declare no conflicts of interest.

## Supporting information




**Supporting File 1**: adhm71725‐sup‐0001‐SuppMat.docx.

**Supporting File 2**: adhm71725‐sup‐0001‐VideoS1.

## Data Availability

The code produced for this paper is available in a GitHub repository (https://github.com/simobone19). The data supporting the findings of this study are available from the corresponding author upon request.

## References

[1] F. Bray , M. Laversanne , and H. Sung , “Global Cancer Statistics 2022: GLOBOCAN Estimates of Incidence and Mortality Worldwide for 36 Cancers in 185 Countries,” CA: A Cancer Journal for Clinicians 74 (2024): 229–263, 10.3322/caac.21834. 38572751

[2] P. Krzyszczyk , A. Acevedo , and E. J. Davidoff , “The Growing Role of Precision and Personalized Medicine for Cancer Treatment,” Technology 06 (2018): 79–100, 10.1142/s2339547818300020. PMC6352312 30713991

[3] V. Subbiah , G. Curigliano , and J. K. Sicklick , “Cancer Treatment Paradigms in the Precision Medicine Era,” Nature Medicine 31 (2025): 3609–3611, 10.1038/s41591-025-03711-w. 40394190

[4] Q. Wang , X. Shao , and Y. Zhang , “Role of Tumor Microenvironment in Cancer Progression and Therapeutic Strategy,” Cancer Medicine 12 (2023): 11149–11165, 10.1002/cam4.5698. PMC10242329 36807772

[5] A. Morales and R. E. Hershberger , “Variants of Uncertain Significance,” Circulation: Genomic and Precision Medicine 11 (2018): 002169, 10.1161/CIRCGEN.118.002169. PMC5999032 29848615

[6] M. Kapałczyńska , T. Kolenda , and W. Przybyła , “2D and 3D Cell Cultures—A Comparison of Different Types of Cancer Cell Cultures,” Archives of Medical Science 14 (2018): 910–919, 10.5114/aoms.2016.63743. PMC6040128 30002710

[7] Z. N. Abbas , A. Z. Al‐Saffar , S. M. Jasim , and G. M. Sulaiman , “Comparative Analysis Between 2D and 3D Colorectal Cancer Culture Models for Insights Into Cellular Morphological and Transcriptomic Variations,” Scientific Reports 13 (2023): 18380, 10.1038/s41598-023-45144-w. PMC10603139 37884554

[8] I. Van Zundert , B. Fortuni , and S. Rocha , “From 2D to 3D Cancer Cell Models—The Enigmas of Drug Delivery Research,” Nanomaterials 10 (2020): 1–30, 10.3390/nano10112236. PMC7696259 33187231

[9] V. Foglizzo , E. Cocco , and S. Marchiò , “Advanced Cellular Models for Preclinical Drug Testing: From 2D Cultures to Organ‐on‐a‐Chip Technology,” Cancers 14 (2022): 3692, 10.3390/cancers14153692. PMC9367322 35954355

[10] T. Ho and R. Msallam , “Tissues and Tumor Microenvironment (TME) in 3D: Models to Shed Light on Immunosuppression in Cancer,” Cells 10 (2021): 831, 10.3390/cells10040831. PMC8067689 33917037

[11] K. Sharma , S. Dey , R. Karmakar , and A. K. Rengan , “A Comprehensive Review of 3D Cancer Models for Drug Screening and Translational Research,” Cancer Innovation 3 (2024): 102, 10.1002/cai2.102. PMC11212324 38948533

[12] P. Mu , S. Zhou , and T. Lv , “Newly Developed 3D in Vitro Models to Study Tumor–Immune Interaction,” Journal of Experimental & Clinical Cancer Research 42 (2023): 81, 10.1186/s13046-023-02653-w. PMC10074642 37016422

[13] S. B. Han , J. K. Kim , G. Lee , and D. H. Kim , “Mechanical Properties of Materials for Stem Cell Differentiation,” Advanced Biosystems 4 (2020): 2000247, 10.1002/adbi.202000247. 33035411

[14] N. D. Evans and E. Gentleman , “The Role of Material Structure and Mechanical Properties in Cell–Matrix Interactions,” Journal of Materials Chemistry B 2 (2014): 2345–2356, 10.1039/c3tb21604g. 32261407

[15] A. Passaniti , H. K. Kleinman , and G. R. Martin , “Matrigel: History/Background, Uses, and Future Applications,” Journal of Cell Communication and Signaling 16 (2022): 621–626, 10.1007/s12079-021-00643-1. PMC9733768 34463918

[16] T. Bhattacharjee , C. J. Gil , and S. L. Marshall , “Liquid‐Like Solids Support Cells in 3D,” ACS Biomaterials Science & Engineering 2 (2016): 1787–1795, 10.1021/acsbiomaterials.6b00218. 33440476

[17] N. Contessi Negrini , A. Franchi , and S. Danti , “Biomaterial‐Assisted 3D in Vitro Tumor Models: From Organoid Towards Cancer Tissue Engineering Approaches,” Cancers 15 (2023): 1201, 10.3390/cancers15041201. PMC9953828 36831542

[18] Y. Jo , N. Choi , K. Kim , H. J. Koo , J. Choi , and H. N. Kim , “Chemoresistance of Cancer Cells: Requirements of Tumor Microenvironment‐Mimicking In Vitro Models in Anti‐Cancer Drug Development,” Theranostics 8 (2018): 5259–5275, 10.7150/thno.29098. PMC6276092 30555545

[19] O. Tartagni , A. Borók , and E. Mensà , “Microstructured Soft Devices for the Growth and Analysis of Populations of Homogenous Multicellular Tumor Spheroids,” Cellular and Molecular Life Sciences 80 (2023): 93, 10.1007/s00018-023-04748-1. PMC10020259 36929461

[20] S. Petralla , F. De Chirico , and A. Miti , “Epigenetics and Communication Mechanisms in Microglia Activation With a View on Technological Approaches,” Biomolecules 11 (2021): 1–29, 10.3390/biom11020306. PMC7923060 33670563

[21] S. N. Bhatia and D. E. Ingber , “Microfluidic Organs‐on‐Chips,” Nature Biotechnology 32 (2014): 760–772, 10.1038/nbt.2989. 25093883

[22] F. Formaggio , E. Saracino , and M. Barbalinardo , “A 3D‐bioprinted Dermal‐Like Scaffold Incorporating Fibroblasts and DRG Neurons to Investigate Peripheral Nerve Regeneration,” Journal of Materials Chemistry B 13 (2025): 7034–7047, 10.1039/d4tb02823f. 40421636

[23] M. H. C. Y. H. Huang , Q. L. Guo , Y. X. Chen , L. J. Zhang , Z. X. Chen , and X. Z. Wang , “Interleukin‑10 Promotes Primary Rat Hepatic Stellate Cell Senescence by Upregulating the Expression Levels of p53 and p21,” Molecular Medicine Reports 17 (2018): 5700–5707, 10.3892/mmr.2018.8592. PMC5866012 29436649

[24] D. I. Wallace and X. Guo , “Properties of Tumor Spheroid Growth Exhibited by Simple Mathematical Models,” Frontiers in Oncology 3 (2013): 51, 10.3389/fonc.2013.00051. PMC3598098 23508803

[25] A. P. Browning , J. A. Sharp , and R. J. Murphy , “Quantitative Analysis of Tumour Spheroid Structure,” Elife 10 (2021): 73020, 10.7554/eLife.73020. PMC8741212 34842141

[26] D. Das , R. Preet , and P. Mohapatra , “5‐Fluorouracil Mediated Anti‐Cancer Activity in Colon Cancer Cells Is Through the Induction of Adenomatous Polyposis Coli: Implication of the Long‐Patch Base Excision Repair Pathway,” DNA Repair 24 (2014): 15–25, 10.1016/j.dnarep.2014.10.006. PMC4830146 25460919

[27] J. Friedrich , C. Seidel , R. Ebner , and L. A. Kunz‐Schughart , “Spheroid‐Based Drug Screen: Considerations and Practical Approach,” Nature Protocols 4 (2009): 309–324, 10.1038/nprot.2008.226. 19214182

[28] C. G. Williams , H. J. Lee , T. Asatsuma , R. Vento‐Tormo , and A. Haque , “An Introduction to Spatial Transcriptomics for Biomedical Research,” Genome Medicine 14 (2022): 68, 10.1186/s13073-022-01075-1. PMC9238181 35761361

[29] B. T. Grünwald and R. Khokha , “Tumour Tissue: Heterogeneous, but Not Disordered,” Nature Reviews Cancer 25 (2025): 821–822, 10.1038/s41568-025-00852-5. 40659926

[30] S. B. Huang , D. Chou , and Y. H. Chang , “Development of a Pneumatically Driven Active Cover Lid for Multi‐Well Microplates for Use in Perfusion Three‐Dimensional Cell Culture,” Scientific Reports 5 (2015): 18352, 10.1038/srep18352. PMC4680883 26669749

[31] R. Sharma , M. R. Perez , and V. A. da Silva , “3D bioprinting Complex Models of Cancer,” Biomaterials Science (2023): 3414–3430, 10.1039/d2bm02060b. 37000528

[32] L. Zhao , J. Xiu , and Y. Liu , “A 3D Printed Hanging Drop Dripper for Tumor Spheroids Analysis Without Recovery,” Scientific Reports 9 (2019): 19717, 10.1038/s41598-019-56241-0. PMC6928160 31873199

[33] E. M. Langer , B. L. Allen‐Petersen , and S. M. King , “Modeling Tumor Phenotypes In Vitro With Three‐Dimensional Bioprinting,” Cell Reports 26 (2019): 608–623.e6, 10.1016/j.celrep.2018.12.090. PMC6366459 30650355

[34] R. H. Utama , L. Atapattu , and A. P. O'Mahony , “A 3D Bioprinter Specifically Designed for the High‐Throughput Production of Matrix‐Embedded Multicellular Spheroids,” Iscience 23 (2020): 101621, 10.1016/j.isci.2020.101621. PMC7567958 33089109

[35] D. G. Nguyen , J. Funk , and J. B. Robbins , “Bioprinted 3D Primary Liver Tissues Allow Assessment of Organ‐Level Response to Clinical Drug Induced Toxicity In Vitro,” PLoS One 11 (2016): 0158674, 10.1371/journal.pone.0158674. PMC4936711 27387377

[36] X. Wei , Y. Wu , K. Chen , L. Wang , and M. Xu , “Embedded Bioprinted Multicellular Spheroids Modeling Pancreatic Cancer Bioarchitecture Towards Advanced Drug Therapy,” Journal of Materials Chemistry B 12 (2024): 1788–1797, 10.1039/d3tb02913a. 38268422

[37] B. Ayan , D. Nyoung Heo , and Z. Zhang , “Aspiration‐Assisted Bioprinting for Precise Positioning of Biologics,” Science Advances 6 (2020): aaw5111, 10.1126/sciadv.aaw5111. PMC7060055 32181332

[38] B. Ayan , N. Celik , and Z. Zhang , “Aspiration‐Assisted Freeform Bioprinting of Pre‐Fabricated Tissue Spheroids in a Yield‐Stress Gel,” Communications Physics 3 (2020): 183, 10.1038/s42005-020-00449-4. PMC7695349 33251340

[39] M. H. Kim , Y. P. Singh , and N. Celik , “High‐Throughput Bioprinting of Spheroids for Scalable Tissue Fabrication,” Nature Communications 15 (2024): 10083, 10.1038/s41467-024-54504-7. PMC11582690 39572584

[40] A. Johnson , P. K. S. Ng , and M. Kahle , “Actionability Classification of Variants of Unknown Significance Correlates With Functional Effect,” NPJ Precision Oncology 7 (2023): 67, 10.1038/s41698-023-00420-w. PMC10349825 37454202

[41] J. Chen , L. Larsson , A. Swarbrick , and J. Lundeberg , “Spatial Landscapes of Cancers: Insights and Opportunities,” Nature Reviews Clinical Oncology 21 (2024): 660–674, 10.1038/s41571-024-00926-7. 39043872

[42] A. Engberg , C. Stelzl , O. Eriksson , P. O'Callaghan , and J. Kreuger , “An Open Source Extrusion Bioprinter Based on the E3D Motion System and Tool Changer to Enable FRESH and Multimaterial Bioprinting,” Scientific Reports 11 (2021): 21547, 10.1038/s41598-021-00931-1. PMC8566469 34732783

[43] S. Bonetti , github‐Bioprinting, (n.d.). December 17, 2024, https://github.com/simobone19.

[44] Addgene Plasmid #127909 “SB100X in pCAG Globin pA″ Sleeping Beauty Transposase Expression Vector Driven by the CAG Promoter”, http://n2t.net/addgene:127909, (last visited on January 8^th^, 2026).

[45] A. R. Strom , R. J. Biggs , and E. J. Banigan , “Hp1α is a Chromatin Crosslinker That Controls Nuclear and Mitotic Chromosome Mechanics,” Elife 10 (2021): 63972, 10.7554/eLife.63972. PMC8233041 34106828

[46] Addgene Plasmid #60507 “pSBtet‐Pur SB‐transposon with inducible SfiI cloning site for GOI (contains firefly luciferase) and constitutive expression of rtTA and puromycin resistance gene,” http://n2t.net/addgene:60507, (last visited on January 8^th^, 2026).

[47] E. Kowarz , D. Löscher , and R. Marschalek , “Optimized Sleeping Beauty Transposons Rapidly Generate Stable Transgenic Cell Lines,” Biotechnology Journal 10 (2015): 647–653, 10.1002/biot.201400821. 25650551

[48] Addgene Plasmid #24130 “pUltra‐hot 3rd generation Lentiviral vector for bi‐cistronic expression of mCherry and the gene of interest,” http://n2t.net/addgene:24130, (last visited on January 8^th^, 2026).

[49] F. Farabegoli , M. Govoni , E. Spisni , and A. Papi , “Epigallocatechin‐3‐Gallate and 6‐OH‐11‐O‐hydroxyphenanthrene Limit BE(2)‐C Neuroblastoma Cell Growth and Neurosphere Formation in Vitro,” Nutrients 10 (2018): 1141, 10.3390/nu10091141. PMC6164794 30135355

[50] A. Surucu , D. de Biase , and C. Ricci , “Beta‐Catenin Alterations in Postchemotherapy Yolk Sac Tumor, Postpubertal‐Type with Enteroblastic Features,” Modern Pathology 37 (2024): 100513, 10.1016/j.modpat.2024.100513. 38763421

[51] D. De Biase , G. Acquaviva , and M. Visani , “Molecular Diagnostic of Solid Tumor Using a next Generation Sequencing Custom‐designed Multi‐gene Panel,” Diagnostics 10 (2020): 250, 10.3390/diagnostics10040250. PMC7236002 32340363

