# Supplementary material for: A Hybrid Bioprinting–Microfabrication Approach to Controllable Multitype Spheroid Arrays for Modeling Tumor Heterogeneity
Source: Adv Healthc Mater. 2026 Sep 20;15(38):e71725. doi: 10.1002/adhm.71725 (PMC13674128; doi:10.1002/adhm.71725)
Supplement: Supplementary file 1 [file ADHM-15-0-s001.docx]

Supporting Information

**A hybrid bioprinting–microfabrication approach to controllable multitype spheroid arrays for modeling the tumor heterogeneity**

*Simone Bonetti,^1^* Eva Clemente,^1^ Emanuela Mensà,^2^ Marco de Maria,^2^ Fulvia Farabegoli,^2^ Emanuela Saracino,^3^ Barbara Bortolani,^4^ Emanuela Marcelli,^4^ Renato Brandimarti,^2^ Dario de Biase,^2^ and Giampaolo Zuccheri^2,5,6^*.*

1) Institute for the Study of Nanostructured Materials, National Research Council of Italy, Bologna, Italy
2) Department of Pharmacy and Biotechnology, University of Bologna, Bologna, Italy.

*Emanuela Saracino*

3) Institute for the Organic Synthesis and Photoreactivity, National Research Council of Italy, Bologna, Italy.
4) eDIMES Lab – DIMEC, Università di Bologna, Policlinico S. Orsola-Malpighi, Bologna, Italy.
5) Interdepartmental Center for Industrial Research on Health Science and Technologies (CIRI-SDV), Alma Mater Studiorum Università di Bologna, Via Piero Gobetti, 87, 40129 Bologna, Italy.
6) S3 Center, Institute of Nanoscience of the National Research Council at the University of Modena, Italy.

For correspondence: E-mail: [simone.bonetti@cnr.it](mailto:simone.bonetti@cnr.it); giampaolo.zuccheri@unibo.it

**Supplementary methods**

**1.1 MSA printing with multiple syringes**

This protocol has been developed to print arrays with different solutions. All printing macros and files can be found on the GitHub repository[see ref. 43 in the main text].

To begin, reset the Z offset values in the config-override.g and tpost1 files. **Step1:** After making these changes, restart the system to apply proper initialization. Perform the homing procedure for the bioprinter, then move the XY axes to the centre of the plate and set the Z axis to 152 mm. Mount the syringes in the bioprinter and ensure they are in contact with the syringe pump plungers for proper alignment. **Step2:** Start the Z height comparison protocol to measure and adjust the offsets between the different tools using one of the following macros: Z-offset T0 vs T1 (for two tools), Z-offset T0 vs T1-T2 (for three tools), Z-offset T0 vs T1-T2-T3 (for four tools)^1^. Enter the measured values into the config-override.g and tpost1 files. Once updated, reset the machine and perform homing again. **Step3:** Access Control Point Coordinates and launch TAMV [TAMV site] on the Raspberry Pi (the number of active tools will be detected automatically from config.g). Move the tool changer to the position required for aligning the Control Point (CP) using the macro “control point coordinates” and set the Z axis to 152 mm. Confirm the CP on the TAMV GUI and proceed with axis alignment. Allow sufficient time for the system to save the calibration data before closing TAMV. Verify that the updated values have been correctly saved in config-override.g, then close TAMV. After tool calibration, it is necessary to calibrate Tip T0 relative to the microstructured well. **Step4:** First, experimentally determine the Z value of Tip T0 when in contact with the plate (Z-T0_Plate). The theoretical Z position corresponding to the top of the microwell device is: ZtopDeviceTheo = Z-T0_Plate + 6.9 mm. To verify the correct coordinates, move the tip to a safe Z position (ZtopDeviceTheo + 30 mm) and position it over the top-right microwell of well 1 using the command: G1 X117.000 Y80.000 F1000.00. Then, lower the tip to ZtopDeviceTheo. Using the bottom camera, check the experimental X (X_exp) and Y (Y_exp) coordinates, adjusting until the tip is aligned with the microwell. Lower the tip until it lightly touches the surface of the device to determine the experimental ZtopDevice (ZtopDeviceExp). Calculate the XY shift using: X shift = X_exp - 117 (theoretical X), Y shift = Y_exp - 80 (theoretical Y). The Z deposition height is experimentally determined and set as: Z_deposition = ZtopDeviceExp - 0.65 mm. Input the X and Y offset values into the LabVIEW software and generate the G-code accordingly.

**1.2 MSA printing with one syringe**

This protocol has been developed to print arrays of spheroids using a single solution. In this study, it was used for two purposes: printing the spheroid array and treating the pre-formed spheroids after a certain number of days in culture with another solution. This protocol includes only specific steps from the previously described procedure, namely Step 1 and Step 4, as tool alignment is not required when using a single tool. Specifically, for printing the spheroid array, the deposition height is set to: Z_deposition = Z_topDeviceExp - 0.65 mm. For treating the previously printed spheroids, the deposition height is adjusted to: Z_deposition = Z_topDeviceExp - 0.45 mm. This prevents scratching or stressing the spheroids within the microwell.

**1.3 GUI of the Software for Micro-seeding the Device**

**Panel A – "Choose Wells"**

The **"Generate G-code"** button allows the operator to generate the G-code based on the selected settings. The **12 large buttons** in the central part represent the wells of a **12-well plate**, enabling the operator to select the well where micro-seeding will occur. At the bottom, the operator can choose the file path for saving the G-code and specify the file name. The **"Stop"** button halts the software (see Figure S13, left panel).

**Panel B – "Settings"**

This panel contains the settings and parameters for array printing in each well selected in **Panel A**. The **"Printing Parameters"** and **"Velocities"** sections include the general parameters required to generate the G-code. In the **"Printing Parameters"** section, the **"Distance between microwells"** defines the spacing between microwells in the device. The **"H movement between wells"** specifies the height the tip must reach when moving between different wells, while the **"H movement between microwells"** determines the height required for movements between microwells within the same well. The **"H deposition in microwell"** represents the height at which the tip deposits the solution into the microwell. The **"H device for spheroid formation"** indicates the height at which the needle tip touches the upper surface of the device. The **"Extrusion"** parameter defines the displacement (in mm) imposed on the extrusion piston of the printing tool to deposit the solution in each microwell. The **"Extrusion Multiplier full well"** is the multiplier applied to the **"Extrusion"** parameter for the entire printing protocol, while the **"Extrusion Velocity well"** sets the extrusion velocity applied to all extrusion commands.

In the **"Velocity"** section, different velocity parameters can be adjusted to generate the G-code. **"XY Velocity Between Microwells"** refers to the velocity set for movement between microwells within the same device, while **"XY Velocity Between Wells"** defines the velocity for movement between different wells. **"Z Velocity Microwell"** is the velocity assigned to vertical movements within a microwell, whereas **"Z Velocity Well"** specifies the velocity for vertical movements within a well. **"Time to Wait in Microwell"** represents the time (in milliseconds) that the needle tip remains stationary after depositing the solution in the microwell. In the **"XY Shift to Apply to Theoretical Value"** section, experimental shifts can be applied to the theoretical XY values. **"X Center Experimental Shift"** represents the X-axis shift mentioned in **Step 4** of the **Materials and Methods** section titled **"MSA Printing with Multiple Syringes,"** while **"Y Center Experimental Shift"** refers to the Y-axis shift. In the **"Proportional Micro-seeding"** section, activating the mode with the **"Active Multiplier"** button enables the printing of an array with proportional columns, allowing different numbers of cells per column. The **"Proportional T-1/0-F0/1"** button adjusts the proportional direction. If the button is set to **true**, the number of cells decreases from right to left, leaving the last column empty. If set to **false**, the number of cells increases from right to left, leaving the first column empty (Figure S12). **"Extrusion Multiplier Each Row"** is a proportional multiplier applied during proportional micro-seeding. The **"Z Multiplier"** can be used in all modes and modifies the **H deposition in microwells** for each printed column. It maintains the set value at the central column, decreasing proportionally by **0.1 mm** for each column to the left and increasing proportionally by **0.1 mm** for each column to the right (see Figure S13, right panel). The **"Two Population"** button activates this function. The parameters **"First Column," "Second Column Group,"** and **"Third Column Group"** respectively indicate the multipliers applied to the extrusion value for different columns. The **"Rows Seeding"** section enables differential seeding for rows grouped in sets of three. The **"Second Seed"** button activates this protocol. The parameters **"First Rows," "Second Rows,"** and **"Third Row"** respectively indicate the multipliers applied to the extrusion value for different rows (Figure S13).

**Supplementary Figures**

**Figure S1**: a) Microstructured wells created by fitting thin microstructured devices in multi-well culture plates. b) The devices were fabricated using both poly-dimethylsiloxane (PDMS, device shown on the left side of the image) and agarose (device shown on the right side of the image). The microstructured devices were replica molded from c) microstructured 3D-printed resin molds.

**Figure S2:** Representative images of HCT116 spheroids grown in (a) PDMS and (b) agarose MSA (scale bar = 750 µm). Images were obtained by brightfield light-transmission microscopy.

**Figure S3**: Comparison of the sizes of the homogeneous HCT116 spheroids obtained on different devices within the same 12-well plate with twelve 81-microwell devices that were fully seeded within the same operation. Each device was seeded with an increasing number of cells across its columns (same number of seeded cells within the rows). All the resulting spheroids in 4 devices (ranging from the beginning to the end of the operation) were measured and averaged across the rows. The measurements were done after 5 days of culture.

**Figure S4**: Viability test of HCT116 exposed to different concentrations of Histodenz™ for 30 min or 0 min (plated immediately after mixing with the Histodenz™ stock solution). HCT116 cells were grown in 2D for 3 days after treatment with Histodenz™ to test long-term effects on cell growth, and then the MTT cell vitality assay was performed. Cells remained in an approximately 1:3 dilution of the stated concentration of Histodenz for their first day in culture to allow adhesion to the plate before medium exchange. 20% Histodenz™ implies that the medium density is significantly higher than the cells’ density (and cells tend to float).

**Figure S5:** HCT cells seeded on the bottom of the microwell (a) with a Z value of 450 µm and (b) with a Z value of 1050 µm

**Figure S6.** Fluorescence micrographs of HCT116 cells stained for a live/dead assay: with fluorescein diacetate (FDA**,** green for live cells) and propidium iodide (red, for dead cells) under different conditions: slides were prepared by spreading cells from a suspension. (a) before printing, without Histodenz™ in the cell solution; (b) before printing, with cell medium containing Histodenz™; (c) after printing, collected in an Eppendorf tube with Histodenz™ in the cell medium; (d) in the microwell, after printing with Histodenz™ in the cell medium. Scale bar: 100 µm.

|  |  |
| --- | --- |
|  |  |

Figure S7. Illustration of the process of making composite images of the spheroids in the MSA from an array of micrographs: the example of Figure 4d. 25 individual micrographs of the microwells hosting the cell spheroids were collected by scanning the culture plate on an inverted microscope (with a 4X objective) to cover the entire area of the array of microwells. For selected rows (in this case, rows g and h), the image portions of the centers of the microwells were cut and arranged on a dark background to facilitate the readers’ observation. The four composites were then stacked to show the growth during the culture. The first column of the arrays (from the left) only contains a small amount of debris that does not grow during the incubation time.

**Figure S8**: Average spheroid area per MSA (MSAs at positions 1, 4, 8, 12, out of a full 12-device plate printing) after 5 days of culture. Each MSA contains mixed-spheroids arrays (as in Figure 5c) composed of linearly varying amounts of HCT-GFP and HCT-mCherry cells for a total seeding of 330 cells/microwell. (n = 81 per condition; error bars = SD).

**Figure S9**: The response of HCT116 cells after 72 hours of treatment with 5-FU while in a 2D culture (blue markers) or in a 3D culture (green markers). The curve fits were obtained with Hill’s equation. The IC50 was estimated to be about 8 µM for 2D cultures and about 25 µM for 3D cultures.

**Figure S10**: Growth profile of HCT116 spheroids seeded with the bioprinter with increasing cell numbers (in the same multiwell device) and treated for 3 days with 40 nM Paclitaxel after 3 days of growth to allow the formation of the spheroids. On the left: the size of the treated spheroids is compared with the size at 3 days of growth and at 6 days of growth of untreated spheroids (in alternate multiwell devices with the same geometry and seeding densities). 40 nM corresponds to the IC50 concentration as determined by us on 3D cultures of HCT116 of a single size that were seeded manually in microwell devices [see ref. 19 of the main text]. On the right: the spheroid-by-spheroid ratio of sizes at 6 days compared to sizes at 3 days.

**Figure S11**: MSA growth in extracellular matrix. Spheroids of a range of sizes (increasing left to right) have been seeded in agarose microwells to make HCT116 MSA. One day after cell seeding, a layer of approximately 20% (V/V) Matrigel**^®^** was spread over the microwell devices. Growth was continued for 3 more days, and then 25 μM of 5-FU was added to one MSA, while another was left untreated and continued its growth in culture medium. The first row of the micrograph gallery above shows an example row of one MSA with spheroids of increasing size.

Figure S12: Tool‑tip positioning repeatability after multiple tool‑change events. After completion of the TAMV alignment procedure, tool‑0 and tool‑1 were alternately moved to a nominal coordinate corresponding to the center of the field of view of the camera. Following each tool change, an image was acquired to quantify the positioning repeatability error of the tool tip. Ten measurements were collected in total (five per tool, alternated sequentially). Although only the first two points are representative of the most complex printing protocol used in this study (two seeding events performed with tool‑0 and tool‑1), the full dataset represents a stress test of the system, showing a maximum positional deviation of 23 µm along the X axis and 12 µm along the Y axis after ten consecutive tool‑change cycles.

**Figure S13**: GUI of the custom-written software for micro-seeding the device.

| Extruded Volume (nl) | 40 | 80 | 120 | 160 | 200 | 240 | 280 | 320 |
| --- | --- | --- | --- | --- | --- | --- | --- | --- |
| Expected number of cells | 40 | 80 | 120 | 160 | 200 | 240 | 280 | 320 |
| Number of cells | 33 | 88 | 124 | 154 | 207 | 247 | 287 | 329 |
| Standard Deviation | 13 | 14 | 21 | 27 | 25 | 36 | 29 | 34 |
| Relative Deviation (%) | 32.5 | 17.5 | 17.5 | 16.9 | 12.5 | 15.0 | 10.4 | 10.6 |

**Table S1**: Estimation of the dispensing errors in the cell seeding. Different numbers of cells were extruded in the microwells (in a medium supplemented with 14% Histodenz™) and counted with brightfield microscopy (also see plot in Figure 3a). The relative standard deviation progressively decreases with printing higher numbers of cells, as expected. Small microscale fluctuations in the local cell density in solution and a higher volume dispensing error for small travel distances in the syringe pumps are possible causes of this.

**Table S2**: Variant allele frequency of the different variants detected by NGS in the analyzed specimens
